# Remarkable structural transformations of actin bundles are driven by their initial polarity, motor activity, crosslinking, and filament treadmilling

**DOI:** 10.1371/journal.pcbi.1007156

**Published:** 2019-07-09

**Authors:** Aravind Chandrasekaran, Arpita Upadhyaya, Garegin A. Papoian

**Affiliations:** 1 Department of Chemistry and Biochemistry, University of Maryland, College Park, Maryland, United States of America; 2 Institute for Physical Science and Technology, University of Maryland, College Park, Maryland, United States of America; 3 Department of Physics, University of Maryland, College Park, United States of America; Boston University, UNITED STATES

## Abstract

Bundled actin structures play a key role in maintaining cellular shape, in aiding force transmission to and from extracellular substrates, and in affecting cellular motility. Recent studies have also brought to light new details on stress generation, force transmission and contractility of actin bundles. In this work, we are primarily interested in the question of what determines the stability of actin bundles and what network geometries do unstable bundles eventually transition to. To address this problem, we used the MEDYAN mechano-chemical force field, modeling several micron-long actin bundles in 3D, while accounting for a comprehensive set of chemical, mechanical and transport processes. We developed a hierarchical clustering algorithm for classification of the different long time scale morphologies in our study. Our main finding is that initially unipolar bundles are significantly more stable compared with an apolar initial configuration. Filaments within the latter bundles slide easily with respect to each other due to myosin activity, producing a loose network that can be subsequently severely distorted. At high myosin concentrations, a morphological transition to aster-like geometries was observed. We also investigated how actin treadmilling rates influence bundle dynamics, and found that enhanced treadmilling leads to network fragmentation and disintegration, while this process is opposed by myosin and crosslinking activities. Interestingly, treadmilling bundles with an initial apolar geometry eventually evolve to a whole gamut of network morphologies based on relative positions of filament ends, such as sarcomere-like organization. We found that apolar bundles show a remarkable sensitivity to environmental conditions, which may be important in enabling rapid cytoskeletal structural reorganization and adaptation in response to intracellular and extracellular cues.

## Author summary

Actin bundles are a salient feature of the cellular cytoskeleton offering structural integrity to the cell in addition to playing a key functional role in processes such as intracellular transport, mechanosensing and locomotion. Even though bundles have been studied extensively for years, a comprehensive picture of bundle stability is still lacking. In this study, we used MEDYAN, a publicly available software package for simulating active polymer networks at single-molecule resolution, to study how bundle’s initial geometry, molecular motors and crosslinkers determine the corresponding lifetime and eventual fate of the bundle. We found that unipolar bundles, where all filaments point in the same direction, are significantly more stable compared with mixed-polarity (apolar) bundles. We also show that while high myosin activity results in transition to aster-like morphology, increased treadmilling causes network collapse and fragmentation. Overall, bundle dynamics and morphological transformations are largely driven by the tug-of-war between filament treadmilling and myosin activity, leading in some cases to very interesting architectures, such as sarcomere-like organization. We propose that the ability of apolar bundles, in particular, to undergo remarkable structural transformations in response to perturbations of the environment may be crucial for cytosplasmic reorganization.

## Introduction

Understanding emergent behaviors in the actin cytoskeleton is important, since key biological functions such as cellular growth, division, and motility depend on cytoskeletal dynamics. Actin networks are transient and malleable within a single cell, constantly forming and remodeling various micro-architectures at different cellular locations. One salient class of such actin structures are actin bundles [1,2], which appear both in the cell body as stress fibers [3–5], or in specialized cellular processes such as filopodia [6–8], stereocilia [9–11] and microvilli [11–13]. The formation and functionality of bundles is spatially and temporally regulated by various proteins that interact with actin, including nucleation factors [5,14], crosslinkers [15,16] and molecular motors [17]. Depending on the cellular context and specific influence of actin regulatory proteins, different bundle structures emerge [18–20], with distinct functional roles as elaborated next.

First, owing to the polarity of individual actin filaments, an actin bundle may be unipolar, apolar, sarcomeric or of graded polarity (S1 Fig in S1 Text) [1], being tethered at either one or both ends via a cross-linker or a molecular motor. Cellular structures of unipolar bundles are ubiquitous across living cells: they aid in cytoplasmic streaming in pollen tubes [21–23] as well as in fungal hypha formation [24]. Unipolar bundles are found in animal cells in filopodia [25] and proximal dorsal stress fibers [5,26]. These structures are usually crosslinked but do not contain myosin as active crosslinkers *in vivo* [27]. However, there is evidence for myosin minifilaments, which are polymers of 10–30 myosin units [28], walking through tracks of unipolar bundles [29] under *in vitro* conditions. Non-muscle myosin II-A isoform minifilaments (NMII-A) [30] incorporate into dorsal stress fibers when these stress fibers are connected to transverse arcs [27]. Recently, NMI has also been implicated in filopodial force generation in neural growth cones [31]. Other actin bundle organizations, such as apolar bundles, underlie the portions of stress fibers near the cell center in fibroblasts [32], in sections of contractile rings [33,34] and in sections of ventral and transverse stress fibers. Composite apolar bundles are mostly found as two unipolar precursor bundles interacting with one another due to filament sliding in response to NMII-A activity [29]. Finally, more finely organized polarity arrangements are found in sarcomeric ordering, which has great significance in generating contractility of stress fibers [1,2].

The large variety of actin fibers and their distinct functional roles have instigated a growing interest in how various factors, such as crosslinking activity and minifilament concentrations, affect the dynamics and stability of bundles having unipolar and apolar organization. Another salient property of many actin bundles namely, non-sarcomeric contractility [35–37], has also been a focal topic of experimental and computational studies. An agent based model was employed by Zemel et al. to study one dimensional unipolar and apolar bundles of 10μm length under different concentrations of motors moving according to prescribed force-velocity relations [38]. They found that apolar bundles readily undergo sliding motions resulting in internal sorting. However, the lack of spatial details in their model did not allow predictions of the stability of bundle morphology under the influence of myosin. Dasanayake et al. [39] simulated actin bundles of 10μm length, modeled in 2D, considering explicit filament stretching and bending in addition to minifilament stretching forces in a confined volume. They found that apolar bundles exert higher wall stresses than parallel filaments.

Nevertheless, it is still unclear how actin bundles behave at long time scales under a wide range of crosslinker and myosin concentrations, especially if they were to be modeled in three spatial dimensions. The latter point should be emphasized because studying actin bundles in 3D is crucial for reconciling with the phenomenology of bundles observed under *in vivo* cellular conditions. Recent studies in three dimensions by Kim and coworkers indicated that filament polarity plays an important role in bundle formation from disordered actin networks [40] and also in tension generation [40,41]. To shed further light on the mechanochemistry of actin bundles, in this work we set out to establish the fundamental principles that govern the stability of two fundamental bundle organizations, namely, purely unipolar and apolar bundles.

Understanding conditions that either stabilize or destabilize various bundle geometries will shed light on the basic principles that govern cytoskeletal organization, bringing insights into complex *in vivo* processes, such as cytoplasmic disassembly [42] and actin network turnover [43–45]. For example, stability of untethered bundles is likely to depend on their internal polarity structure as well as crosslinker and myosin motor conditions. Furthermore, cells modulate actin filament turnover through a host of mechanisms such as filament severing [46–48], branching [1,49] and capping [50,51]. As a result, *in vivo* turnover rates are orders of magnitude faster than those commonly observed in *in vitro* experiments [47,52–54]. In general, actin turnover plays a critical role in stress relaxation of entangled actin networks at longer timescales. For example, skeletal myosin is known to fluidize actin networks at very low mole ratios (myosin head to total actin mole ratio, M:A 0.0039) [55]. On the other hand, myosin‘s catch bond behavior can lead to long residence times resulting in actin networks maintaining large internal stresses at high myosin concentrations. In addition, transient passive crosslinkers arrest network configurations and prevent relaxation, however, crosslinker unbinding events allow for slow reconfiguration dynamics [56,57]. The crosstalk between filament turnover and crosslinker mechanokinetics has not been comprehensively studied under a wide range of conditions. Hence, it is also necessary to explore stability of bundles under a broad set of treadmilling rates.

To address the above outlined problems, we have used MEDYAN (MEchanochemical Dynamics of Active Networks) mechanochemical force field [58] to study the stability and dynamics of untethered actin bundles under a diverse set of polarity arrangements and levels of α-actinin crosslinker, myosin minifilament and treadmilling conditions. MEDYAN is further elaborated in the Methods section below. Our main finding is that unipolar bundles preserve bundle morphology at a wider range of crosslinker and, myosin concentrations than apolar bundles, because the latter experience a morphological instability due to being more susceptible to myosin induced intra-bundle filament sliding and shearing. We also show that three salient microarchitectures eventually emerge when simulating various untethered bundles under different conditions—bundles, asters and bundle-aster hybrids. Asters are filamentous networks exhibiting radial polarity sorting, with barbed ends clustered towards the aster center. We also investigated how the resulting phase diagrams depend on the speed of treadmilling. We found that network catastrophes, characterized by poorly crosslinked low density networks with obscure morphologies, occur when crosslinking cannot keep up with filament extension. Overall, our studies demonstrate that while a stand-alone apolar bundle is stable under a significantly narrower set of conditions compared to unipolar bundles, this geometric arrangement can serve as an important precursor to rich network remodeling phenomena such as global polarity sorting and sarcomeric organization.

## Methods

MEDYAN is a mechanochemical force field for simulating active matter, including cytoskeletal networks. It deeply integrates chemical and transport dynamics with network mechanics, treating these phenomena on equal footing. MEDYAN has emerged from earlier efforts to model actin bundle growth in filopodia [59–63], where both active and passive transport were shown to critically influence growth dynamics [60,62,63]. MEDYAN’s time evolution is based on alternating reaction-diffusion and mechanical equilibration steps, where the former events are stochastically generated according to the *next reaction method* [64], while the conjugate gradient approach is used to achieve mechanical equilibration [58]. This propagation scheme takes advantage of the wide timescale separation between slow chemical processes and fast mechanical equilibration speeds within sub-micron length scale volumes containing a portion of an actin network [65]. Furthermore, MEDYAN can effectively model actin filament polymerization processes as well as explicit α-actinin (crosslinker), myosin minifilament (motor) binding and unbinding events, in addition to myosin walking and mechanochemical feedbacks such as catch and slip bond behaviors.

In contrast to MEDYAN, other cytoskeletal modeling strategies, such as Cytosim (Cytoskeletal Simulation) [66,67], AFINES (Active Filament Network Simulation) [68], and the model by Kim and coworkers [41] rely on the Langevin dynamics of cytoskeletal components, explicitly simulating thermal undulations at the expense of significant diminution of computational efficiency. Among these models, MEDYAN’s treatment of general reaction-diffusion processes is most comprehensive (in particular, with regard to G-actin’s diffusion and reactions). In addition, MEDYAN’s mechanical potentials are the most elaborate, for example, having an analytical excluded-volume potential representing steric repulsions and also complex dihedral angle potentials at the dendritic actin network branch points. A detailed comparison of different cytoskeletal modeling approaches can be found in Popov et al. (in particular, see Table S1 of [58]).

In MEDYAN, the reaction volume is divided into voxels based on the Kuramoto length [58,69]. Diffusing molecules are assumed to be uniformly mixed within each voxel and can diffuse from one voxel to another. Actin filaments can polymerize and depolymerize from both plus and minus ends based on experimentally determined rate constants [70]. Filaments that polymerize towards the boundary experience a reduction in polymerization rate based on the Brownian ratchet model [71]. In MEDYAN, the growth propensity for an actin filamentous tip is based on the local, instantaneous concentration of diffusing G-actin in the tip’s neighborhood [58], while, in comparison, actin filaments grow/shrink at a constant rate in Cytosim, [72]. Furthermore, MEDYAN can explicitly account for ATP, ADP.Pi, and ADP bound actin monomeric states, enabling more elaborate simulations of F-actin polymerization dynamics [73,74]. In summary, filament length fluctuations and filament treadmilling can be studied using MEDYAN at the resolution of a single actin monomer. As elaborated below, we found that these fluctuations play an important role in determining whether the bundle stays coherent or undergoes a morphological transformation.

**Fig 1:**
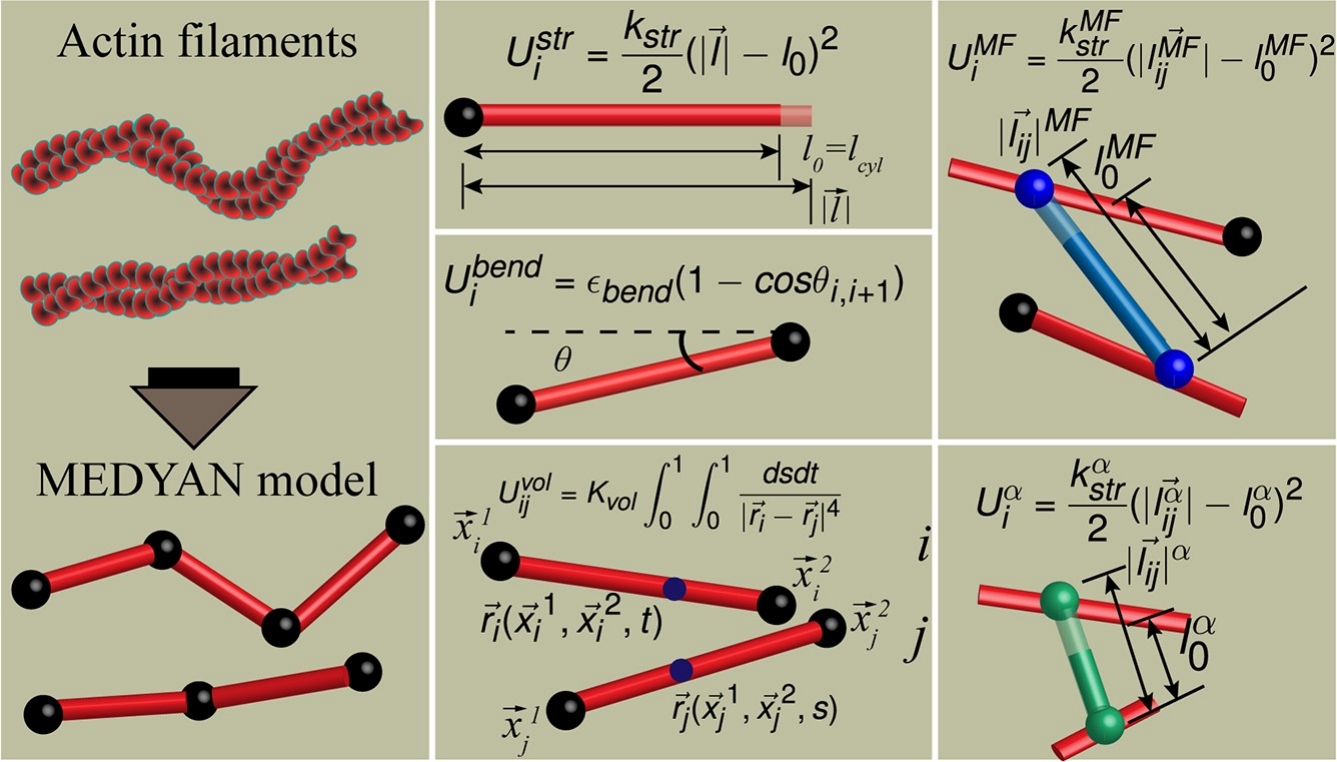
The mechanical model of actin, α-actinin and myosin minifilaments in MEDYAN. Double helix structure of actin filament is represented as a series of cylinders (red) connected together at hinges (black spheres). Stretching (U_i_^str^), bending (U_i_^bend^) and cylinder-cylinder excluded volume (U_ij_^vol^) interactions are elaborated. Crosslinkers (green) and myosin MF (blue) experience stretching potentials (U_i_^α^ and U_i_^MF^ respectively). More details are provided in the Methods section and the Supporting Methods (section 2 in S1 Text).

In MEDYAN, mechanical modeling of actin filaments is based on cylinder units with equilibrium spacing $l_{0}^{m}\ll l_{p}(l_{0}^{m}$ = 108 nm in this study, *lp*-persistence length) connected with neighboring cylinders at flexible hinges. Persistence length of actin was reported from experiments as 17μm [75]. The MEDYAN force-field prevents filaments passing through each other via a novel cylinder-cylinder repulsion potential that is analytical, in contradistinction to the more widely employed technique of other comparable force fields, which relies on finding the closest distance between two cylinders that is used to compute their mutual repulsion [58]. Various MEDYAN mechanical potentials, such as intra-cylinder stretching and inter-cylinder bending are shown in Fig 1. α-actinin and myosin molecules that are bound to actin filaments are modeled as springs connecting two actin filament sites within their respective binding distances (α-actinin binding distance is in the range 30–40 nm, and minifilament binding distance is in the range 175–225 nm).

Those reactions that are mechanosensitive, for example, unbinding kinetics of actin binding proteins such as crosslinkers and motors, are influenced by the local instantaneous stresses of the actin network, via corresponding modifications of the reaction rate constants. Motor/crosslinker binding and unbinding are modeled as a single step chemical reaction in MEDYAN, while in other force fields, for example, Cytosim, these processes occur via two elementary steps, comprising of separate reactions for each end of the motor or crosslinker [76]. In MEDYAN, crosslinker unbinding kinetics is modeled as a slip bond while myosin unbinding kinetics is modeled as a catch bond based on the parallel cluster model [77]. The motor walking rate is given by a linear force-velocity relationship for motor walking events. Further aspects of mechanical equilibration and mechanochemical feedback loops in MEDYAN as well as the implementation details of the chemical model are provided in Supporting Information (Section 2 in S1 Text).

Initial structures for all our simulations were based on 2 μm long actin bundles, comprising 30 actin filaments that correspond to *in vivo* stress fibers [32], where the internal arrangements of filament polarities were in either unipolar or apolar geometries. Filaments were initially placed on a hexagonal lattice with a spacing of 35 nm as found in experiments [78,79]. Bundles were modeled with α-actinin and NMIIA minifilaments that can bind and unbind from actin filaments. Bundles were simulated at 7 different concentrations of α-actinin (α-actinin to total actin mole ratio referred to henceforth as α:A 0.01, 0.05, 0.1, 0.25, 0.4, 0.6, 0.8) and 6 different concentrations of myosin (myosin head to total actin mole ratio referred to henceforth as M:A 0.0225, 0.045, 0.09, 0.18, 0.225, 0.675). 8 trajectories, each 2000 seconds long, were generated for each of the 6x7 = 42 mole ratio pairs (α:A, M:A) studied.

In addition, we also studied how the speed of filament treadmilling influences bundle stability. Myosin mole ratios of 0.0225 0.045, 0.225 and 0.675 were considered at α:A 0.01, 0.1 and 0.4 to investigate how all observed non-treadmilling morphologies behave under different treadmilling conditions. Treadmilling speed was varied by simultaneously altering polymerization and depolymerization rates at both filament ends by the same factor χ. As a reference, χ = 1.0 corresponds to the *in-vitro* treadmilling rate. We chose the following χ values, (0.1, 0.3, 0.6, 1.0, 3.0, 6.0, and 10.0), hence mimicking treadmilling speeds that are both slower and faster than the *in vitro* rate. As a technical detail, in this work we have developed a flexible volume protocol that permits expanding and contracting the reaction volume along the X-axis, which allows avoiding artificial boundary effects on the bundle major axis in a computationally efficient manner. This technique is further elaborated in Supporting Information (Section 2.5 in S1 Text). 7 trajectories were generated for each combination of the myosin mole ratio, α-actinin mole ratio and χ factor.

In order to understand the underlying morphologies sampled, we devised a hierarchical clustering scheme. To carry out structural clustering of obtained bundle configurations, we first computed the distributions of plus end—plus end (Dis^++^), minus end—minus end (Dis^—^), and plus end—minus end (Dis^+-^) Euclidean distances. Jensen Shannon divergences [80] between each of the 42 mole ratios taken pair-wise were used to construct initial -condition -specific dissimilarity matrices (S12 Fig in S1 Text). The complete linkage method [81] results in a hierarchical cluster, which we visualized as dendrograms (shown below). Supporting Methods Section 2.4.1 in S1 Text provides further details on the clustering algorithm.

## Results

### Long timescale morphologies of actomyosin bundles are primarily determined by their initial polarity and the myosin concentration

**Fig 2:**
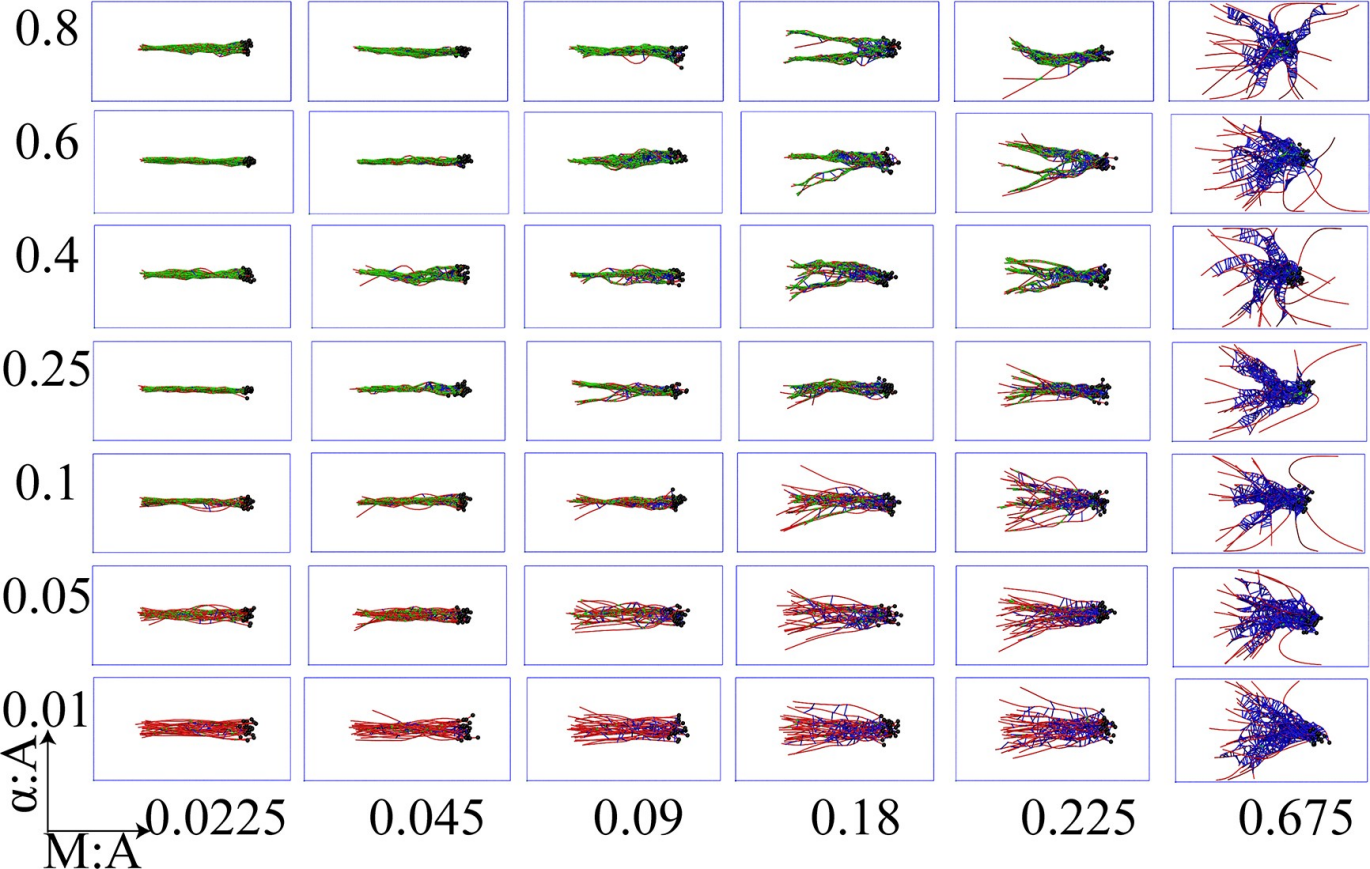
Representative snapshots show network morphologies from simulations of unipolar bundles under different crosslinker and myosin mole ratios with respect to the actin concentration. Each panel shows a steady state network configuration of actin (colored in red) along with bound myosin minifilaments (blue) and α–actinin (green). Mole ratios of myosin and α-actinin with respect to actin are held at values mentioned along abscissa and ordinate of the grid respectively.

**Fig 3:**
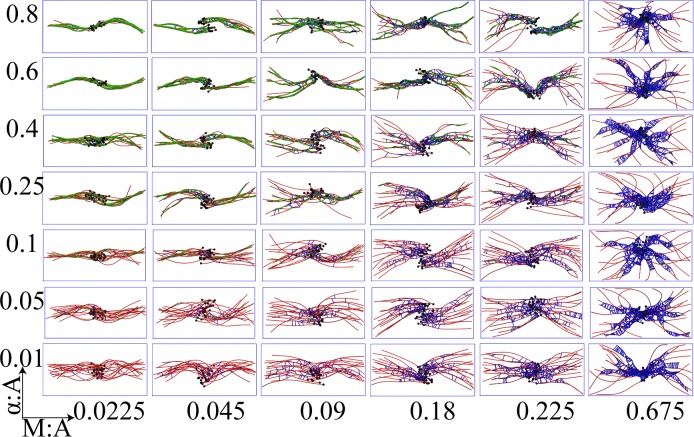
Representative snapshots show network morphologies from simulations of apolar bundles under different crosslinker and myosin mole ratios with respect to the actin concentration. Each panel shows a steady state network configuration of actin (colored in red) along with bound myosin minifilaments (blue) and α–actinin (green). Mole ratios of myosin and α-actinin with respect to actin are held at values mentioned along abscissa and ordinate of the grid respectively.

A wide array of steady state network morphologies emerge when non-treadmilling bundles with unipolar initial organization (non-treadmilling-BUInit) and apolar initial organization (non-treadmilling-BAInit) were simulated under a broad set of α-actinin and myosin concentrations (Figs 2 and 3 and S1 and S2 Movies). A trajectory is considered to have reached steady state when the network’s radius of gyration reaches a stationary value (see S10 Fig in S1 Text). We found that for any combination of α-actinin and myosin ratios, marked differences are observed between steady state network morphologies of apolar and unipolar bundles. More specifically, antiparallel filaments show a strong tendency to mutually slide in response to myosin activity as a consequence of the latter being unidirectional walkers (S2 Movie). To quantitatively characterize the resulting network morphologies, we applied a novel structure-based clustering analyses that we have developed in this work (see the Methods section and the Supporting Information, Section 2.4.1 in S1 Text), which revealed dominant network morphologies preferred under various conditions, as elaborated below.

**Fig 4:**
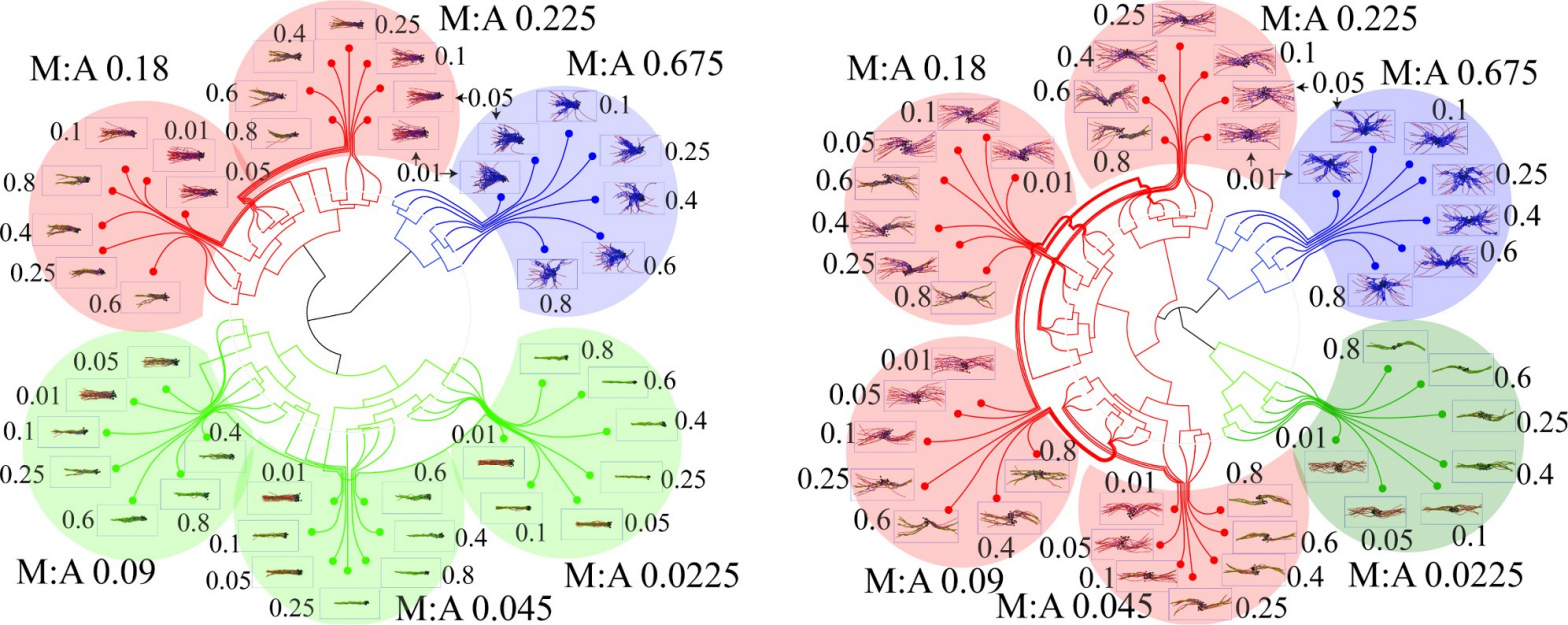
Dendrograms illustrating clustering of different resultant actin network morphologies from either unipolar (left) or apolar (right) bundle initial configurations. Distributions of distances between minus-minus, minus-plus and plus-minus ends were used to construct dissimilarity matrices for both unipolar and apolar cases. Agglomerative cluster trees were encoded from the above-mentioned dissimilarity matrices and then drawn as dendrograms. The three largest clusters are shown in red, blue and green along with representative final snapshots. α:A values are indicated close to the snapshots while M:A values are indicated for each sub-cluster. Clades corresponding to bundle-like configurations are colored in green while aster-like configurations are colored in blue. Intermediate states that do not resemble either network morphologies are colored in red.

The resulting dendrograms reveal three broad clusters closest to the root, colored in green, red and blue, pointing to three major network morphologies (Figs 2–4): bundle-like (BL), aster-like (AL) and aster-bundle intermediate (ABI) states. We also considered an alternative morphology classification technique based on network radial distribution devised by Freedman et al. [82] (see S3 Fig in S1 Text) and a combination of nematic order and shape parameters (see S2 Fig and Supplementary Methods 2.4.2 and 2.4.3 in S1 Text). Both order parameters delineated well the bundled morphologies from the aster-like morphologies. However, the hierarchical classification strategy introduced in this work was also able to identify the intermediate bundle-aster morphologies.

Some reflection on the internal structure of these dendrograms shows that within each initial polarity arrangement, NMIIA concentration is the main driver of the resulting network morphology (see Fig 4). Specifically, the same highest level clusters are formed from configurations with similar M:A ratios, while finer-grained additional clustering is determined by other factors, such as the crosslinker (α-actinin) concentration. The effect of myosin is primary because at high motor concentrations inter-filament distances become significantly widened outside of the α-actinin binding compatibility zone of 30–40 nm. This, in turn, crucially depletes the network of crosslinker binding sites (S4 Fig in S1 Text), hence, greatly diminishing parallel alignment among actin filaments and, subsequently, destabilizing the bundle phase.

It is interesting to compare our finding of the depletion of crosslinkers with increasing myosin concentration with the recent simulations that studied sorting of two different crosslinkers along an actin bundle. Freedman et al. have established that a significant length difference between two actin binding proteins, when combined with a specific range of the filament bending moduli, can result in spatial sorting of crosslinkers along a bundle [83]. Here, we found that active myosin walking may significantly increase overall inter-filament separation, thereby reducing the number of sites available for crosslinker binding in the bundles studied here, which presumably should have important implications for the sorting phenomenon in the presence of molecular motors.

When simulations were started with unipolar initial conditions, bundle-like states were stable when M:A ≤ 0.09 (21/42 cases = 50% of all M:A/α:A mole ratios studied). On the other hand, apolar initial arrangements result in stable bundle-like states only in ~20% (7/42) of the cases (i.e. when M:A = 0.0225). Thus, unipolar bundles are stable under a significantly wider range of conditions than their apolar counterparts. We tracked this difference primarily to myosin activity in apolar bundles giving rise to two thin polarity sorted sub-bundles that are together much longer than the initial bundle length and, furthermore, mutually interact via their barbed ends (see Figs 3 and 4). The resulting thinner bundles are more susceptible to bending deformations than the corresponding unipolar bundle. Consequently, at the time scale of about 30 minutes probed in this work, unipolar and apolar bundles arrive at different metastable morphologies despite being under the same crosslinker and myosin conditions. Presumably, if these systems were to be ergodic, then bundles are expected to eventually evolve to identical steady state configurations regardless of the initial polarity arrangement. However, as shown in our previous works [84,85], cytoskeletal dynamics maybe sufficiently glassy such that only metastable states are reachable over laboratory timescale.

Under very high myosin activity (M:A = 0.675), both unipolar and apolar bundles undergo a morphological collapse, preferring radially symmetric aster-like structures (the last column in Figs 2 and 3). We note in passing that in cells, asters are primarily found in microtubule networks as radially symmetric structures with filament plus ends spatially clustered together [86,87]. Actin networks are also expected to form radially polarity sorted asters [88]. Such structures have been found in *in vitro* treadmilling actin networks subject to skeletal muscle myosin (M:A 0.1), fascin and myosin [89] or just skeletal muscle myosin (M:A 0.02) alone [90,91], with the filament plus ends oriented towards the center of the aster.

The difference in threshold myosin ratios between skeletal muscle myosin and NMIIA for the onset of aster-like structures is partly explained by the increased processivity of muscle myosin. Actin asters were observed *in-vitro* when cytoskeletal structures were destabilized using Cytochalasin D [42]. Recently, they were shown to be essential in fission yeast cells during fusion [92,93]. Fission cell actin asters are considered to be formed due to Fus1 nucleators and multimerization of Myo51 and Myo52 [93]. Overall, we found that experimentally observed salient network morphologies of treadmilling networks can also be sampled in our simulations under non-treadmilling conditions.

### Initial polarity arrangement plays a key role in the evolution of the treadmilling bundle morphology

Next, we investigated the combined effect due to network turnover and mechanokinetics (crosslinker, and NMIIA) by including actin filament polymerization and depolymerization processes in bundle simulations analogous to the systems discussed above. In the treadmilling study, steady state was defined when the average filament length fluctuations reach their stationary values (see S11 Fig in S1 Text). To systematically modulate filament treadmilling, we varied polymerization and depolymerization rates at both barbed and pointed ends by a factor χ between 0.1 and 10.0, where lower χ values cause slower treadmilling. We modeled a reaction volume having dimensions of 4 μm x 1.5 μm x 1.5 μm, with an initial total actin concentration of 5 μM, where the simulation box can expand and contract along the major axis based on the instantaneous bundle length (more details are provided in the Supplementary Information, Section 2.5).

We investigated both unipolar and apolar bundles at M:A mole ratios of 0.0225, 0.09, 0.225 and 0.675, whereas α:A was sampled at 0.01, 0.1 and 0.4. These values were carefully chosen to capture the different salient network morphologies manifested under non-treadmilling conditions (non-treadmilling-BUInit and non-treadmilling-BAInit), namely, BL, AL and ABI states. 7 trajectories, each 2000 seconds long, were generated for each of the 84 triad conditions (α:A, M:A, χ), starting from either unipolar (χ-BUInit) or apolar (χ-BAInit) initial conditions. Similar to non-treadmilling bundles, the above-mentioned classes of network morphologies were determined by clustering trajectories from 84 triads using the same clustering protocol (S12 Fig in S1 text). We classify connected networks with morphologies that do not belong to BL, AL or ABI as either Type A catastrophes if the networks are fragmented into smaller clusters or Type B catastrophes, where filaments in the network are poorly connected (S5–S9 Figs in S1 Text).

We found that these catastrophes emerge from the interplay between inter-filament connectivity and treadmilling. Treadmilling is characterized by a net depolymerization at minus ends and filament growth at the plus ends. In particular, increasing χ leads to a faster rate of filament growth at plus ends (and faster depolymerization at minus ends). In these systems, network stability is assured as long as the newly formed filament segments are effectively crosslinked. Under conditions where the latter does not take place, filament treadmilling dominates the system’s structural evolution, leading to poorly connected networks. When well-structured initial bundle configurations result in a highly fragmented network, we denote such transitions as catastrophes (see S5 Fig in S1 Text). On the other hand, at higher mole ratios of α-actinin or myosin, the rate of inter filament connections is enhanced, which prevents network catastrophes.

Having established how inter-filament connections influence network stability, we next investigated the effect of treadmilling speed. Our clustering analysis indicates that treadmilling networks starting from unipolar initial configurations attain BL and ABI morphologies (i.e. non-aster, non-fragmented morphologies) in 33/84 (~40%) cases, compared to 19/84 (~21%) cases for the apolar initial configurations. On the other hand, the apolar initial arrangements lead to aster-like structures in 39/84 (46%) cases as opposed to 20/84 (24%) cases for the unipolar cases. Taken together, these results demonstrate that treadmilling bundles that were evolved from unipolar initial configurations are less likely to undergo morphological collapse than those evolved from apolar initial configurations.

**Fig 5:**
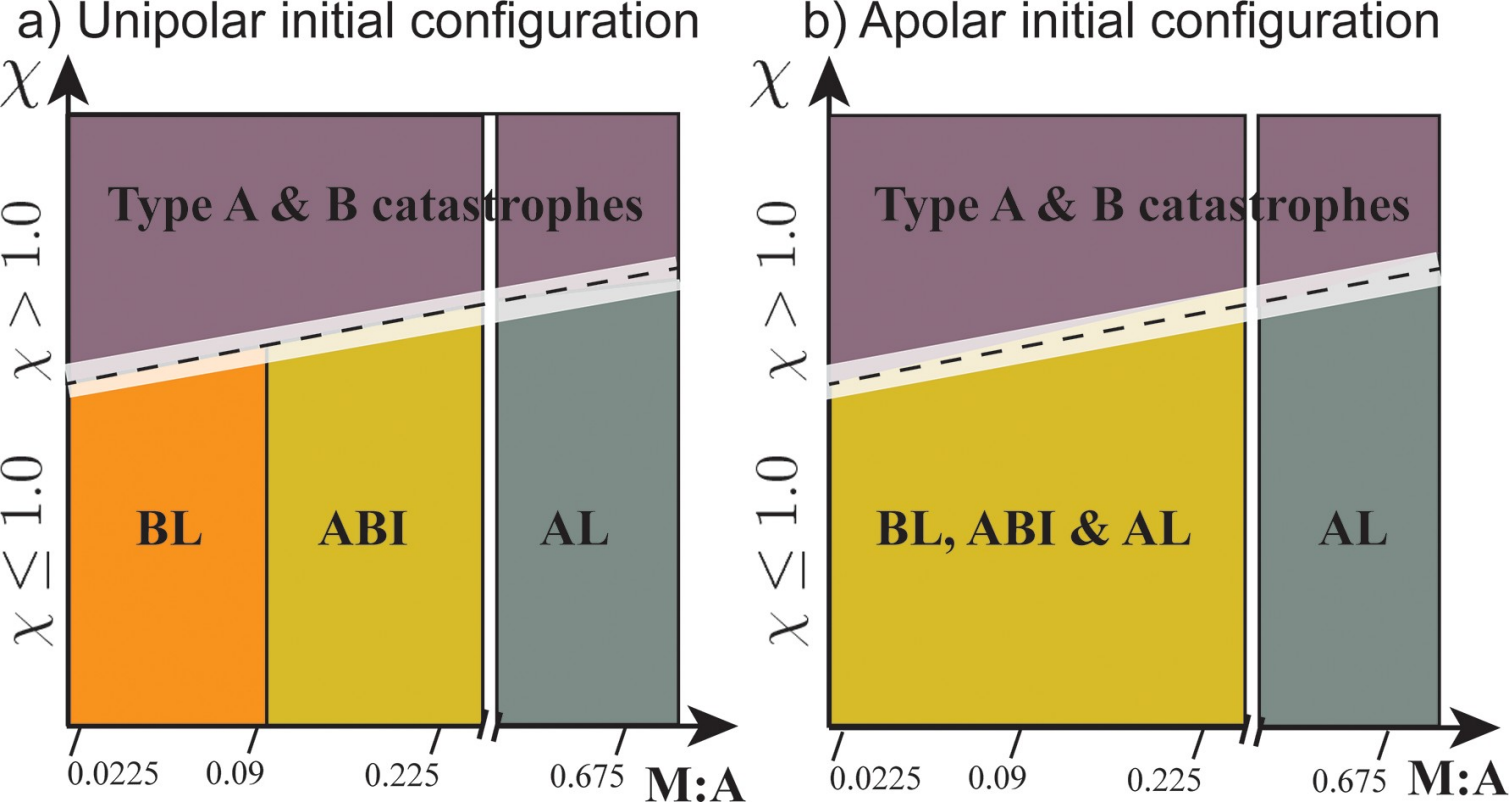
Schematic of preferred network morphologies under different values of triad. Cartoon illustration of dominant network morphologies of bundles expected in **a)** unipolar and **b)** apolar initial configurations at different ranges of treadmilling factor namely χ = 0, 0>χ≤1.0 (0.1, 0.3, 0.6, 1.0) and χ>1.0 (3.0, 6.0, 10.0) Morphologies observed at discrete values of triad along with results from clustering (S5 Fig in S1 Text) were used to propose the map above. Colored voxels are used to represent distinct zones of preferred network morphologies expected. Dotted line represents uncertainty in the boundary. Dotted lines within white bands represent the crosslinker- and myosin-dependent boundaries between stable and unstable networks.

Based on the network morphologies observed at the sampled triad combinations, we suggest the following two phase diagrams, shown in Fig 5, indicating dominant network morphologies as functions of M:A and χ. To justify the choice of these two order parameters, we point out that α-actinin dynamics determine bundle stability in conjunction with myosin, however, affecting only very weakly the final network morphology of stable networks. Fig 5A suggests that treadmilling unipolar networks sample similar network morphologies to the non-treadmilling cases for χ ≤1.0. At larger χ values, network treadmilling dominates as crosslinkers and minifilaments cannot effectively connect filament segments that are formed, leading to network catastrophes (S6–S9 Figs in S1 Text, see snapshots from simulations with χ >1.0) On the other hand, treadmilling apolar bundles lead to aster-like states (under non-catastrophic triads). The BL, ABI morphologies sampled by treadmilling apolar bundles have rich diversity due to the interplay between myosin activity and treadmilling as explained later.

We finally discuss the weak influence of crosslinkers on the morphology of a treadmilling network. At α:A = 0.01 and under low myosin mole ratios (0.0225, and 0.09 shown in Figures S6 and S7), we found that unipolar bundles treadmilling at χ = 1.0 result in network catastrophes. However, significantly increasing crosslinker concentration (e.g. between 10 and 40 folds) can rescue such networks to prefer bundle-like/intermediate morphologies (S3 and S4 Movies). These findings are in qualitative agreement with the observations by Bidone et al [40] that at high crosslinker concentrations bundles form robustly from networks obtained from a wide range of initial orientational biases (at M:A = 0.08).

### Treadmilling apolar bundles attain a diverse set of network morphologies at long time scales

**Fig 6:**
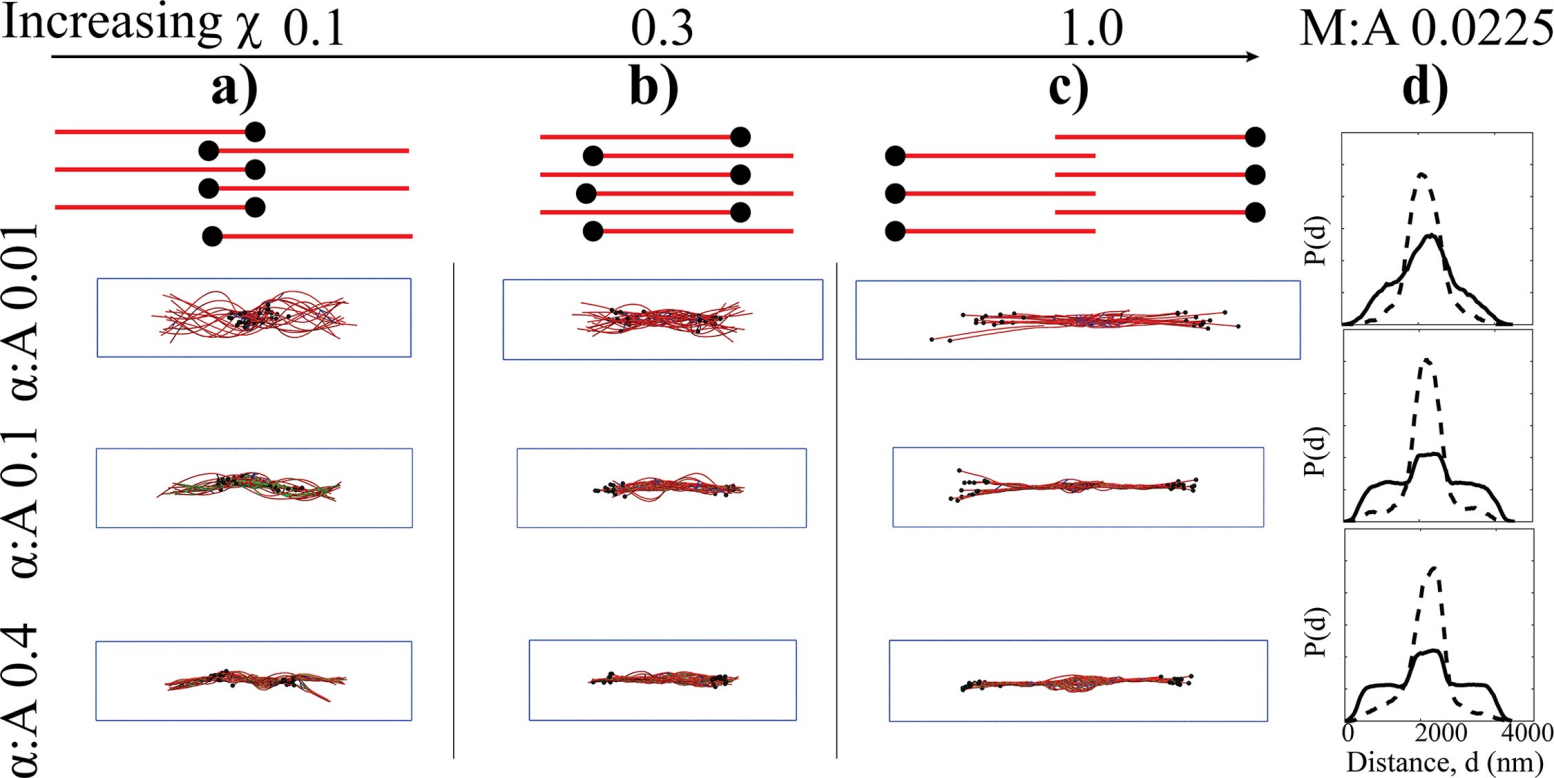
The effects of treadmilling factor (χ) and myosin sliding on the morphologies of BAInit networks are illustrated. Upper panel shows a cartoon of filaments (red) with barbed ends (black tips) representing various orientational arrangements that were realized in our simulations, determined by the interplay between the treadmilling rate and myosin activity. **a-c)** Representative final snapshots from simulations at M:A 0.0225 at various crosslinker mole ratio (α:A) at χ values 0.1 (**a**), 0.3 **(b**) and 1.0 (**c**) are shown. **d)** For networks with χ = 1.0, probability of finding NMIIA (dotted line) and α-actinin (solid line) along bundle axis are plotted.

Under low myosin activity conditions, treadmilling bundles starting from an apolar arrangement generate a remarkably diverse set of final network morphologies, primarily based on how filament barbed ends are spatially localized (see Fig 6). These emergent network geometries arise from the tug-of-war between myosin activity and filament treadmilling. On the one hand, treadmilling apolar bundles are subject to the continuous process of plus end extension and minus end retraction. On the other hand, myosin activity drives mutual sliding of neighboring filaments. If the rate of plus end extension is slower than myosin sliding, myosin activity dominates, leading to two unipolar bundles connected at their plus ends (Fig 6A) or to networks with overlapping plus end segments (Fig 6B). Conversely, under vigorous plus end extension conditions compared with myosin sliding, the network transitions to polarity sorted bundles interacting at their minus ends via myosin (Fig 6C and S5 Movie).

Modulation of χ also controls the overall distribution of myosin minifilaments in such bundles. The analysis of the computed spatial distributions of myosin minifilaments and α-actinin under low myosin concentrations (Fig 6D), indicates that myosin spatially segregates close to pointed ends, characteristic of sarcomeric ordering [18,36,94]. The latter arises when myosin minifilaments interact with minus ends flanked on either side by plus ends.

## Discussion

Actin bundles are important for cellular stability, growth and mechanosensing. While prior experimental and modeling research has primarily focused on bundle formation processes [40,72,95,96], in this work we have addressed the stability and temporal evolution of various bundle configurations. We used MEDYAN, a mechano-chemical forcefield based on molecular principles, to simulate bundle dynamics in 3D. The dimensionality of the model is crucial as filament deformations and mutual interactions are markedly dimension-dependent. In this study, we comprehensively analyzed how α-actinin and myosin influence the stability and morphological transformations of unipolar and apolar actin bundles. We discovered that at time scales of about 2000 seconds, non-treadmilling unipolar bundles are stable under a wider range of crosslinker and myosin mole ratios compared to apolar bundles. At high myosin mole ratios, we observed aster-like states characterized by interacting barbed ends grouped in the center of the cluster with radially emanating pointed ends.

We also investigated how actin turnover affects bundle morphology fates, by developing and applying a simulation protocol that allows moving system boundaries. Our results indicate that treadmilling bundles, both unipolar and apolar, undergo network catastrophes when the network’s ability to form inter-filament connections is insufficient compared to the treadmilling speeds. *In vivo* cytoskeletal networks undergoing fast treadmilling may be able to avoid such undesired fragmentation using additional mechanisms such as filament capping and actin filament nucleators.

Interestingly, at high myosin concentrations, even quick treadmilling does not rescue the network from transitioning to aster-like configurations. On the other hand, at low myosin activity, initially apolar bundles explore a diverse spectrum of network organizations, which we attributed to the tug-of-war between minifilament activity and filament treadmilling. Under certain conditions, interesting, biologically relevant architectures emerge, such as sarcomeric-like organization. We are not aware of prior models that resulted in the spontaneous assembly of sarcomeric arrangements without imposing spatial restrictions on crosslinkers. In particular, previous attempts to reproduce sarcomeric distribution of treadmilling apolar networks relied on various assumptions, such as preferential binding of passive crosslinkers near plus ends [18] or considered systems with both plus and minus end directed motors [97].

Finally, we reflect on the biological consequences that follow from this work. We found that treadmilling bundles with apolar initial configuration are poised to undergo a remarkable morphological response to the perturbations of the environment, such as alterations of myosin activity or treadmilling rates. On the one hand, this level of sensitivity to parameters might be potentially detrimental to the overall stability of the cellular actin network. On the other hand, if only a small fraction of the cytoskeleton is organized as apolar bundles, the latter can sensitively respond to various signaling cues that affect the local concentrations of actin binding proteins. Thus, we propose that apolar bundles might be crucial to cytoskeletal robustness and adaptation in scenarios that demand drastic structural reorganization. The ability to rapidly change network morphology might be important in certain cellular functions where force production or rapid cellular reorganization are necessary. Overall, the optimal choice of bundle architecture should be determined by the specific cellular processes that it affects: for example, in the case of cargo transport [98] or protrusive growth [99], a structurally stable unipolar bundle would be the optimal choice, while contractile elements that require frequent reorganization would be more robust when apolar bundles are incorporated into their architectures [5,100].

In summary, we simulated actin bundles in 3D, while explicitly accounting for excluded volume interactions, diffusion of actin, crosslinker and NMIIA proteins, and numerous chemical and mechanical processes that enhance the model’s realism. We systematically studied the influences of initial bundle polarity, concentrations of myosin and α-actinin and the network turnover rate, finding a remarkably rich palette of bundle evolution trajectories, from stable bundle states to asters and sarcomeric organizations. In future works, additional effects may be considered, such as actin filaments transiently tethering to the substrate and nucleation of filaments via formins or Arp2/3, which will bring us closer to achieving a more complete understanding of bundle dynamics under *in vivo* conditions.

## Supporting information

S1 Text Twelve supplemental figures, and additional details on methods are provided in this document. (DOCX)

S1 Movie Representative trajectories of non-treadmilling unipolar networks at α:A = 0.1 with varying myosin mole ratios (0.0225, 0.18, and 0.675). Actin filaments (red) with plus ends marked as black spheres are shown with bound crosslinkers represented as green dumbbells. Myosin minifilaments are shown in blue.
(MP4)

S2 Movie Representative trajectories of non-treadmilling apolar networks at α:A = 0.1 with varying myosin mole ratios (0.0225, 0.18, and 0.675). Actin filaments (red) with plus ends marked as black spheres are shown with bound crosslinkers represented as green dumbbells. Myosin minifilaments are shown in blue.
(MP4)

S3 Movie Representative trajectories to elucidate effect of crosslinker mole ratio on unipolar bundles treadmilling based on in-vitro polymerization and depolymerization kinetics (χ = 1.0) with M:A 0.0225. Actin filaments (red) with plus ends marked as black spheres are shown with bound crosslinkers represented as green dumbbells. Myosin minifilaments are sown in blue. Reaction volumes expand and contract per protocol described in Supporting Methods 2.5.
(MP4)

S4 Movie Representative trajectories to elucidate effect of crosslinker mole ratio on unipolar bundles treadmilling based on in-vitro polymerization and depolymerization kinetics (χ = 1.0) with M:A 0.09. Actin filaments (red) with plus ends marked as black spheres are shown with bound crosslinkers represented as green dumbbells. Myosin minifilaments are sown in blue. Reaction volumes expand and contract per protocol described in Supporting Methods 2.5.
(MP4)

S5 Movie Representative trajectories showing spatial segregation of myosin minifilaments (M:A = 0.0225) in apolar bundles treadmilling at χ = 1.0 at different crosslinker mole ratios (0.01, 0.1, and 0.4). Actin filaments (red) with plus ends marked as black spheres are shown with bound crosslinkers represented as green dumbbells. Myosin minifilaments are sown in blue. Reaction volumes expand and contract per protocol described in Supporting Methods 2.5.
(MP4)

## References

[1] Skau CT, Waterman CM. Specification of Architecture and Function of Actin Structures by Actin Nucleation Factors. Annu Rev Biophys. 2015;44: 285–310. 10.1146/annurev-biophys-060414-034308 26098516 PMC6301004

[2] Tojkander S, Gateva G, Lappalainen P. Actin stress fibers—assembly, dynamics and biological roles. J Cell Sci. 2012;125: 1855–1864. 10.1242/jcs.098087 22544950

[3] Sanger JW, Sanger JM, Jockusch BM. Differences in the stress fibers between fibroblasts and epithelial cells. J Cell Biol. 1983;96: 961–9. 10.1083/jcb.96.4.961 6339529 PMC2112337

[4] Livne A, Geiger B. The inner workings of stress fibers—from contractile machinery to focal adhesions and back. J Cell Sci. 2016;129: 1293–1304. 10.1242/jcs.180927 27037413

[5] Hotulainen P, Lappalainen P. Stress fibers are generated by two distinct actin assembly mechanisms in motile cells. J Cell Biol. 2006;173: 383–394. 10.1083/jcb.200511093 16651381 PMC2063839

[6] Hashimoto Y, Skacel M, Adams JC. Roles of fascin in human carcinoma motility and signaling: Prospects for a novel biomarker? Int J Biochem Cell Biol. 2005;37: 1787–1804. 10.1016/j.biocel.2005.05.004 16002322

[7] Jacinto A, Wood W, Balayo T, Turmaine M, Martinez-Arias A, Martin P. Dynamic actin-based epithelial adhesion and cell matching during Drosophila dorsal closure. Curr Biol. 2000;10: 1420–1426. 10.1016/S0960-9822(00)00796-X 11102803

[8] Applewhite, Derek A, Barzik, Melanie, Kojima, Shin-ichiro, Svitkina M, Gertler, Frank B, Borisy GG. Ena/VASP proteins have an anti-capping independent function in filopodia formation. Mol Biol Cell. 2007;18: 2579–2591. 10.1091/mbc.E06-11-0990 17475772 PMC1924831

[9] Lewis GT, Hole W, Biology C, Republic F, Biology C, Tilney MS, et al Preliminary biochemical characterization of the stereocilia and cuticular plate of hair cells of the chick cochlea. J Cell Biol. 1989;109: 1711–1723. 10.1083/jcb.109.4.1711 2677026 PMC2115824

[10] Hudspeth AJ, Jacobs R. Stereocilia mediate transduction in vertebrate hair cells (auditory system/cilium/vestibular system). Proc Natl Acad Sci. 1979;76: 1506–1509. 10.1073/pnas.76.3.1506 312502 PMC383283

[11] Narayanan P, Chatterton P, Ikeda A, Ikeda S, Corey DP, Ervasti JM, et al Length regulation of mechanosensitive stereocilia depends on very slow actin dynamics and filament-severing proteins. Nat Commun. Nature Publishing Group; 2015;6: 1–8. 10.1038/ncomms7855 25897778 PMC4523390

[12] Croce A, Cassata G, Disanza A, Gagliani MC, Tacchetti C, Malabarba MG, et al A novel actin barbed-end-capping activity in EPS-8 regulates apical morphogenesis in intestinal cells of Caenorhabditis elegans. Nat Cell Biol. 2004;6: 1173–1179. 10.1038/ncb1198 15558032

[13] Revenu C, Athman R, Robine S, Louvard D. The co-workers of actin filaments: from cell structures to signals. Nat Rev Mol Cell Biol. 2004;5: 635–646. 10.1038/nrm1437 15366707

[14] Mseka T, Coughlin M, Cramer LP. Graded actin filament polarity is the organization of oriented actomyosin II filament bundles required for fibroblast polarization. Cell Motil Cytoskeleton. 2009;66: 743–753. 10.1002/cm.20403 19544402

[15] Resolution H, Microscopy E, Meyer RK, Aebi U. Bundling of actin filaments by alpha-actinin depends on its molecular length. J Cell Biol. 1990;110: 2013–24. 10.1083/jcb.110.6.2013 2351691 PMC2116144

[16] Xu J, Wirtz D, Pollard TD. Dynamic cross-linking by α-actinin determines the mechanical properties of actin filament networks. J Biol Chem. 1998;273: 9570–9576. 10.1074/jbc.273.16.9570 9545287

[17] Medeiros NA, Burnette DT, Forscher P. Myosin II functions in actin-bundle turnover in neuronal growth cones. Nat Cell Biol. 2006;8: 215–226. 10.1038/ncb1367 16501565

[18] Friedrich BM, Fischer-Friedrich E, Gov NS, Safran SA. Sarcomeric pattern formation by actin cluster coalescence. PLoS Comput Biol. 2012;8: 1–10. 10.1371/journal.pcbi.1002462 22685394 PMC3369942

[19] Théry M, Pépin A, Dressaire E, Chen Y, Bornens M. Cell distribution of stress fibres in response to the geometry of the adhesive environment. Cell Motil Cytoskeleton. 2006;63: 341–355. 10.1002/cm.20126 16550544

[20] Voth GA, Bidone TC, Kovar DR, Katkar HH, Aydin F, Applewhite DA, et al Ena/VASP processive elongation is modulated by avidity on actin filaments bundled by the filopodia crosslinker fascin. Mol Biol Cell. 2019;30: mbc.E18-08-0500. 10.1091/mbc.E18-08-0526 30601697 PMC6589784

[21] Shimmen T, Yokota E. Cytoplasmic streaming in plants. Curr Opin Cell Biol. 2004;16: 68–72. 10.1016/j.ceb.2003.11.009 15037307

[22] Yokota E, Vidali L, Tominaga M, Tahara H, Orii H, Morizane Y, et al Plant 115-kDa Actin-Filament Bundling Protein, P-115-ABP, is a Homologue of Plant Villin and is Widely Distributed in Cells. Plant Cell Physiol. 2003;44: 1088–1099. 10.1093/pcp/pcg132 14581634

[23] Yokota E, Shimmen T. The 135-kDa actin-bundling protein from lily pollen tubes arranges F-actin into bundles with uniform polarity. Planta. 1999;209: 264–266. 10.1007/s004250050631 10436230

[24] Bachewich C, Heath IB. Radial F-actin arrays precede new hypha formation in Saprolegnia: implications for establishing polar growth and regulating tip morphogenesis. J Cell Sci. 1998;111: 2005–16. Available: http://www.ncbi.nlm.nih.gov/pubmed/9645948 9645948 10.1242/jcs.111.14.2005

[25] Yang S, Huang FK, Huang J, Chen S, Jakoncic J, Leo-Macias A, et al Molecular mechanism of fascin function in filopodial formation. J Biol Chem. 2013;288: 274–284. 10.1074/jbc.M112.427971 23184945 PMC3537022

[26] Pellegrin S, Mellor H. Actin stress fibres. J Cell Sci. 2007;120: 3491–3499. 10.1242/jcs.018473 17928305

[27] Tojkander S, Gateva G, Schevzov G, Hotulainen P, Naumanen P, Martin C, et al A molecular pathway for myosin II recruitment to stress fibers. Curr Biol. Elsevier Ltd; 2011;21: 539–550. 10.1016/j.cub.2011.03.007 21458264

[28] Verkhovsky AB, Borisy GG. Non-sarcomeric mode of myosin II organization in the fibroblast lamellum. J Cell Biol. 1993;123: 637–652. 10.1083/jcb.123.3.637 8227130 PMC2200132

[29] Reymann A-C, Boujemaa-Paterski R, Martiel J-L, Guerin C, Cao W, Chin HF, et al Actin Network Architecture Can Determine Myosin Motor Activity. Science (80-). 2012;336: 1310–1314. 10.1126/science.1221708 22679097 PMC3649007

[30] Vicente-Manzanares M, Ma X, Adelstein RS, Horwitz AR. Non-muscle myosin {II} takes centre stage in cell adhesion and migration. Nat Rev Mol Cell Biol. Nature Publishing Group; 2009;10: 778–790. 10.1038/nrm2786 19851336 PMC2834236

[31] Sayyad W a., Amin L, Fabris P, Ercolini E, Torre V. The role of myosin-II in force generation of DRG filopodia and lamellipodia. Sci Rep. 2015;5: 7842 10.1038/srep07842 25598228 PMC4648386

[32] Cramer LP, Siebert M, Mitchison TJ. Identification of novel graded polarity actin filament bundles in locomoting heart fibroblasts: Implications for the generation of motile force. J Cell Biol. 1997;136: 1287–1305. 10.1083/jcb.136.6.1287 9087444 PMC2132518

[33] Laporte D, Ojkic N, Vavylonis D, Wu J-Q. α-Actinin and fimbrin cooperate with myosin II to organize actomyosin bundles during contractile-ring assembly. Mol Biol Cell. 2012;23: 3094–3110. 10.1091/mbc.E12-02-0123 22740629 PMC3418305

[34] Craig EM, Dey S, Mogilner A. The emergence of sarcomeric, graded-polarity and spindle-like patterns in bundles of short cytoskeletal polymers and two opposite molecular motors. J Phys Condens Matter. 2011;23: 3741021–10. 10.1088/0953-8984/23/37/374102 21862843 PMC3168571

[35] Kruse K, Jülicher F. Actively contracting bundles of polar filaments. Phys Rev Lett. 2000;85: 1778–1781. 10.1103/PhysRevLett.85.1778 10970612

[36] Kruse K, Jülicher F. Self-organization and mechanical properties of active filament bundles. Phys Rev E—Stat Physics, Plasmas, Fluids, Relat Interdiscip Top. 2003;67: 16 10.1103/PhysRevE.67.051913 12786184

[37] Kreten FH, Hoffmann C, Riveline D, Kruse K. Active bundles of polar and bipolar filaments. Phys Rev E. American Physical Society; 2018;98: 012413 10.1103/PhysRevE.98.012413 30110807

[38] Zemel Assaf, Mogilner A. Motor-induced sliding of microtubule and actin bundles. Phys Chem Chem Phys. 2009;11: 4800 10.1039/b901646e 19506757 PMC2732772

[39] Dasanayake NL, Carlsson AE. Stress generation by myosin minifilaments in actin bundles. Phys Biol. 2013;10: 036006 10.1088/1478-3975/10/3/036006 23595157 PMC3695447

[40] Bidone TC, Jung W, Maruri D, Borau C, Kamm RD, Kim T. Morphological Transformation and Force Generation of Active Cytoskeletal Networks. PLoS Comput Biol. Public Library of Science; 2017;13 10.1371/journal.pcbi.1005277 28114384 PMC5256887

[41] Kim T. Determinants of contractile forces generated in disorganized actomyosin bundles. Biomech Model Mechanobiol. 2015;14: 345–355. 10.1007/s10237-014-0608-2 25103419

[42] Verkhovsky AB, Svitkina TM, Borisy GG. Polarity sorting of actin filaments in cytochalasin-treated fibroblasts. J Cell Sci. 1997;110 (Pt 1: 1693–1704. 9264457 10.1242/jcs.110.15.1693

[43] Wilson CA, Tsuchida MA, Allen GM, Barnhart EL, Applegate KT, Yam PT, et al Myosin II contributes to cell-scale actin network treadmilling through network disassembly. Nature. Nature Publishing Group; 2010;465: 373–377. 10.1038/nature08994 20485438 PMC3662466

[44] Fritzsche M, Lewalle A, Duke T, Kruse K, Charras G. Analysis of turnover dynamics of the submembranous actin cortex. Mol Biol Cell. 2013;24: 757–67. 10.1091/mbc.E12-06-0485 23345594 PMC3596247

[45] van Goor D, Hyland C, Schaefer AW, Forscher P. The role of actin turnover in retrograde actin network flow in neuronal growth cones. PLoS One. 2012;7 10.1371/journal.pone.0030959 22359556 PMC3281045

[46] Bernstein BW, Bamburg JR. ADF/Cofilin: A functional node in cell biology. Trends Cell Biol. Elsevier Ltd; 2010;20: 187–195. 10.1016/j.tcb.2010.01.001 20133134 PMC2849908

[47] Bamburg JR, McGough A, Ono S. Putting a new twist on actin: ADF/cofilins modulate actin dynamics. Trends Cell Biol. 1999;9: 364–370. 10.1016/S0962-8924(99)01619-0 10461190

[48] Bravo-Cordero JJ, Magalhaes MAO, Eddy RJ, Hodgson L, Condeelis J. Functions of cofilin in cell locomotion and invasion. Nat Rev Mol Cell Biol. Nature Publishing Group; 2013;14: 405–417. 10.1038/nrm3609 23778968 PMC3878614

[49] Bugyi B, Carlier M-F. Control of actin filament treadmilling in cell motility. Annu Rev Biophys. 2010;39: 449–470. 10.1146/annurev-biophys-051309-103849 20192778

[50] Shekhar S, Pernier J, Carlier M-F. Regulators of actin filament barbed ends at a glance. J Cell Sci. 2016;129: 1085–1091. 10.1242/jcs.179994 26940918

[51] Winkelman JD, Bilancia CG, Peifer M, Kovar DR. Ena/VASP Enabled is a highly processive actin polymerase tailored to self-assemble parallel-bundled F-actin networks with Fascin. Proc Natl Acad Sci. 2014;111: 4121–4126. 10.1073/pnas.1322093111 24591594 PMC3964058

[52] Chen H, Bernstein BW, Bamburg JR. Regulating actin-filament dynamics in vivo. Trends Biochem Sci. 2000;25: 19–23. 10.1016/S0968-0004(99)01511-X 10637608

[53] Fritzsche M, Li D, Colin-York H, Chang VT, Moeendarbary E, Felce JH, et al Self-organizing actin patterns shape membrane architecture but not cell mechanics. Nat Commun. 2017;8: 17–19. 10.1038/s41467-017-00019-3 28194011 PMC5316839

[54] Muller-Taubenberger A, Diez S, Bretschneider T, Anderson K, Gerisch G. Subsecond reorganization of the actin network in cell motility and chemotaxis. Proc Natl Acad Sci. 2005;102: 7601–7606. 10.1073/pnas.0408546102 15894626 PMC1140407

[55] Humphrey D, Duggan C, Saha D, Smith D, Käs J. Active fluidization of polymer networks through molecular motors. Nature. 2002;416: 413–416. 10.1038/416413a 11919627

[56] Salbreux G, Charras G, Paluch E. Actin cortex mechanics and cellular morphogenesis. Trends Cell Biol. Elsevier Ltd; 2012;22: 536–545. 10.1016/j.tcb.2012.07.001 22871642

[57] Kim T, Gardel ML, Munro ED. Determinants of fluidlike behavior and effective viscosity in cross-linked actin networks. Biophys J. Biophysical Society; 2014;106: 526–534. 10.1016/j.bpj.2013.12.031 24507593 PMC3944977

[58] Popov K, Komianos J, Papoian GA. MEDYAN: Mechanochemical Simulations of Contraction and Polarity Alignment in Actomyosin Networks. PLoS Comput Biol. 2016;12: e1004877 10.1371/journal.pcbi.1004877 27120189 PMC4847874

[59] Lan Y, Papoian G a. The stochastic dynamics of filopodial growth. Biophys J. 2008;94: 3839–3852. 10.1529/biophysj.107.123778 18234810 PMC2367176

[60] Zhuravlev PI, Papoian G a. Molecular noise of capping protein binding induces macroscopic instability in filopodial dynamics. Proc Natl Acad Sci U S A. 2009;106: 11570–11575. 10.1073/pnas.0812746106 19556544 PMC2710665

[61] Zhuravlev PI, Lan Y, Minakova MS, Papoian GA. Theory of active transport in filopodia and stereocilia. Proc Natl Acad Sci. 2012;109: 10849–10854. 10.1073/pnas.1200160109 22711803 PMC3390872

[62] Zhuravlev PI, Der BS, Papoian GA. Design of active transport must be highly intricate: A possible role of myosin and Ena/VASP for G-Actin transport in filopodia. Biophys J. Biophysical Society; 2010;98: 1439–1448. 10.1016/j.bpj.2009.12.4325 20409462 PMC2856189

[63] Zhuravlev PI, Papoian GA. Protein fluxes along the filopodium as a framework for understanding the growth-retraction dynamics. Cell Adh Migr. 2011;5: 448–456. 10.4161/cam.5.5.17868 21975554 PMC3218612

[64] Gibson MA, Bruck J. Efficient Exact Stochastic Simulation of Chemical Systems with Many Species and Many Channels. J Phys Chem A. 2000;104: 1876–1889. 10.1021/jp993732q

[65] Falzone TT, Blair S, Robertson-Anderson RM. Entangled F-actin displays a unique crossover to microscale nonlinearity dominated by entanglement segment dynamics. Soft Matter. Royal Society of Chemistry; 2015;11: 4418–4423. 10.1039/c5sm00155b 25920523

[66] Rickman J, Nédélec F, Surrey T. Effects of spatial dimensionality and steric interactions on microtubule-motor self-organization. Phys Biol. 2019;16: 046004 10.1088/1478-3975/ab0fb1 31013252 PMC7655122

[67] Bun P, Dmitrieff S, Belmonte JM, Nédélec FJ, Lénárt P. A disassembly-driven mechanism explains F-actin-mediated chromosome transport in starfish oocytes. Elife. 2018;7: 1–27. 10.7554/elife.31469 29350616 PMC5788506

[68] Freedman SL, Banerjee S, Hocky GM, Dinner AR. A Versatile Framework for Simulating the Dynamic Mechanical Structure of Cytoskeletal Networks. Biophys J. Biophysical Society; 2017;113: 448–460. 10.1016/j.bpj.2017.06.003 28746855 PMC5529201

[69] Kampen VNG. Stochastic Processes in Physics and Chemistry. Stochastic Processes in Physics and Chemistry. 2007 10.1016/B978-0-444-52965-7.X5000-4

[70] Fujiwara I, Vavylonis D, Pollard TD. Polymerization kinetics of ADP- and ADP-Pi-actin determined by fluorescence microscopy. Proc Natl Acad Sci. 2007;104: 8827–8832. 10.1073/pnas.0702510104 17517656 PMC1885587

[71] Peskin CS, Odell GM, Oster GF. Cellular motions and thermal fluctuations: the Brownian ratchet. Biophys J. 1993; 10.1016/S0006-3495(93)81035-X 8369439 PMC1225726

[72] Letort G, Politi A, Ennomani H, Thery M, Nedelec F, Blanchoin L. Geometrical and Mechanical Properties Control Actin Filament Organization. Biophys J. 2014;106: 568a–569a. 10.1016/j.bpj.2013.11.3153 PMC4446331 26016478

[73] Floyd C, Jarzynski C, Papoian G. Low-dimensional manifold of actin polymerization dynamics. New J Phys. IOP Publishing; 2017;19 10.1088/1367-2630/aa9641

[74] Floyd C, Papoian GA, Jarzynski C. Quantifying Dissipation in Actomyosin Networks. Interface Focus. 2019;9: 201800781–10. 10.1098/rsfs.2018.0078 PMC6501337 31065344

[75] Ott A, Magnasco M, Simon A, Libchaber A. Measurement of the persistence length of polymerized actin using fluorescence microscopy. Phys Rev E. 1993;48 10.1103/PhysRevE.48.R1642 9960868

[76] Nédélec F. Computer simulations reveal motor properties generating stable antiparallel microtubule interactions. J Cell Biol. 2002;158: 1005–1015. 10.1083/jcb.200202051 12235120 PMC2173220

[77] Erdmann T, Albert PJ, Schwarz US. Stochastic dynamics of small ensembles of non-processive molecular motors: The parallel cluster model. J Chem Phys. 2013;139 10.1063/1.4827497 24206337

[78] Meyer RK, Aebi U. Bundling of actin filaments by alpha-actinin depends on its molecular length. J Cell Biol. 1990;110: 2013–2024. 10.1083/jcb.110.6.2013 2351691 PMC2116144

[79] Schaus TE, Taylor EW, Borisy GG. Self-organization of actin filament orientation in the dendritic-nucleation/array-treadmilling model. Proc Natl Acad Sci U S A. 2007;104: 7086–7091. 10.1073/pnas.0701943104 17440042 PMC1855413

[80] Lin J. Divergence Measures Based on the Shannon Entropy. IEEE Trans Inf Theory. 1991;37: 145–151. 10.1109/18.61115

[81] Defays D. An efficient algorithm for a complete link method. Comput J. 1977;20: 364–366. 10.1093/comjnl/20.4.364

[82] Freedman SL, Hocky GM, Banerjee S, Dinner AR. Nonequilibrium phase diagrams for actomyosin networks. Soft Matter. Royal Society of Chemistry; 2018;14: 7740–7747. 10.1039/c8sm00741a 30204203 PMC6192427

[83] Freedman SL, Suarez C, Winkelman JD, Kovar DR, Voth GA, Dinner AR, et al Mechanical and kinetic factors drive sorting of F-actin crosslinkers on bundles. bioRxiv. 2018; 493841 10.1101/493841 PMC6697872 31346091

[84] Komianos JE, Papoian GA. Stochastic Ratcheting on a Funneled Energy Landscape Is Necessary for Highly Efficient Contractility of Actomyosin Force Dipoles. Phys Rev X. 2018;8: 0210061–16. 10.1103/PhysRevX.8.021006

[85] Dobramysl U, Papoian GA, Erban R. Steric Effects Induce Geometric Remodeling of Actin Bundles in Filopodia. Biophys J. Biophysical Society; 2016;110: 2066–2075. 10.1016/j.bpj.2016.03.013 27166814 PMC4939473

[86] Heidemann SR, Kirschner MW. Aster formation in eggs of xenopus laevis. 1975;67: 105–117. 10.1083/jcb.67.1.105 PMC2109591 1236852

[87] Mullins RD. Cytoskeletal mechanisms for breaking cellular symmetry. Cold Spring Harb Perspect Biol. 2010;2: 1–17. 10.1101/cshperspect.a003392 20182610 PMC2827899

[88] Ennomani H, Letort G, Guérin C, Martiel JL, Cao W, Nédélec F, et al Architecture and Connectivity Govern Actin Network Contractility. Curr Biol. 2016;26: 616–626. 10.1016/j.cub.2015.12.069 26898468 PMC4959279

[89] Backouche F, Haviv L, Groswasser D, Bernheim-Groswasser a. Active gels: dynamics of patterning and self-organization. Phys Biol. 2006;3: 264–73. 10.1088/1478-3975/3/4/004 17200602

[90] Soares e Silva M, Depken M, Stuhrmann B, Korsten M, MacKintosh FC, Koenderink GH. Active multistage coarsening of actin networks driven by myosin motors. Proc Natl Acad Sci U S A. 2011;108: 9408–9413. 10.1073/pnas.1016616108 21593409 PMC3111259

[91] Takiguchi K. Heavy meromyosin induces sliding movements between antiparallel actin filaments. J Biochem. 1991;109: 520–7. 10.1093/oxfordjournals.jbchem.a123414 1869506

[92] Arai R, Mabuchi I. F-actin ring formation and the role of F-actin cables in the fission yeast Schizosaccharomyces pombe. J Cell Sci. 2002;115: 887–898. 11870208 10.1242/jcs.115.5.887

[93] Dudin O, Bendezú FO, Groux R, Laroche T, Seitz A, Martin SG. A formin-nucleated actin aster concentrates cell wall hydrolases for cell fusion in fission yeast. J Cell Biol. 2015;208: 897–911. 10.1083/jcb.201411124 25825517 PMC4384723

[94] Hu S, Dasbiswas K, Guo Z, Tee YH, Thiagarajan V, Hersen P, et al Long-range self-organization of cytoskeletal myosin II filament stacks. Nat Cell Biol. 2017;19: 133–141. 10.1038/ncb3466 28114270

[95] Tang H, Laporte D, Vavylonis D. Actin cable distribution and dynamics arising from cross-linking, motor pulling, and filament turnover. Mol Biol Cell. 2014;25: 3006–16. 10.1091/mbc.E14-05-0965 25103242 PMC4230589

[96] Reymann AC, Martiel JL, Cambier T, Blanchoin L, Boujemaa-Paterski R, Théry M. Nucleation geometry governs ordered actin networks structures. Nat Mater. Nature Publishing Group; 2010;9: 827–832. 10.1038/nmat2855 20852617

[97] Craig EM, Dey S, Mogilner A. The emergence of sarcomeric, graded-polarity and spindle-like patterns in bundles of short cytoskeletal polymers and two opposite molecular motors. J Phys Condens Matter. 2011;23: 3741021–10. 10.1088/0953-8984/23/37/374102 21862843 PMC3168571

[98] Courson DS, Nagy S, Ricca BL, Smithback PA, Rock RS, Brawley CM, et al A myosin motor that selects bundled actin for motility. Proc Natl Acad Sci. 2008;105: 9616–9620. 10.1073/pnas.0802592105 18599451 PMC2474510

[99] Lenartowska M, Michalska A. Actin filament organization and polarity in pollen tubes revealed by myosin II subfragment 1 decoration. Planta. 2008;228: 891–896. 10.1007/s00425-008-0802-5 18696106

[100] Costa KD, Hucker WJ, Yin FCP. Buckling of actin stress fibers: A new wrinkle in the cytoskeletal tapestry. Cell Motil Cytoskeleton. 2002;52: 266–274. 10.1002/cm.10056 12112140

