# Supplementary material for: Remarkable structural transformations of actin bundles are driven by their initial polarity, motor activity, crosslinking, and filament treadmilling
Source: PLoS Comput Biol. 2019 Jul 9;15(7):e1007156. doi: 10.1371/journal.pcbi.1007156 (PMC6615854; doi:10.1371/journal.pcbi.1007156)
Supplement: S1 Text — (DOCX) [file pcbi.1007156.s001.docx]

Supporting Information

Remarkable structural transformations of actin bundles are driven by their initial polarity, motor activity, crosslinking, and filament treadmilling

**Aravind Chandrasekaran^1,2^, Arpita Upadhyaya^2,3^, Garegin Papoian^1,2^**

^1^Department of Chemistry and Biochemistry, University of Maryland, College Park, Maryland, United States of America

^2^Institute for Physical Science and Technology, University of Maryland, College Park, Maryland, United States of America

^3^Department of Physics, University of Maryland, Maryland, United States of America

Contents

[**1.** **Supporting Figures** 3](#_Toc10477570)

[**2. Supporting Methods** 10](#_Toc10477571)

[**2.1 Chemical model** 10](#_Toc10477572)

[**2.1.1 Diffusion** 10](#_Toc10477573)

[**2.1.2 α-actinin, myosin minifilament binding and unbinding** 11](#_Toc10477574)

[**2.1.3 Minifilament walking** 11](#_Toc10477575)

[**2.2 Stochastic simulation of actomyosin reaction-diffusion network.** 11](#_Toc10477576)

[**2.2.2 Next reaction method** 12](#_Toc10477577)

[**2.2. Details of mechanical model in MEDYAN** 12](#_Toc10477578)

[**2.2.1. Actin filaments** 12](#_Toc10477579)

[**2.2.2. α-actinin, minifilament model** 13](#_Toc10477580)

[**2.2.2. Boundary** 14](#_Toc10477581)

[**2.3 Mechanochemical coupling** 14](#_Toc10477582)

[**2.3.1. Minifilament walking, binding and unbinding rates** 14](#_Toc10477583)

[**2.3.2 α-actinin unbinding rate** 15](#_Toc10477584)

[**2.4 Trajectory analysis** 16](#_Toc10477585)

[**2.4.1. Clustering analysis** 17](#_Toc10477586)

[**2.4.2. Orientational order parameter (S)** 19](#_Toc10477587)

[**2.4.3. Shape Parameter (Sh)** 19](#_Toc10477588)

[**2.4.4. Probability distribution of linker, motor** 20](#_Toc10477589)

[**2.5 Protocol for flexible volume simulations** 20](#_Toc10477590)

[**References** 21](#_Toc10477591)

# **Supporting Figures**


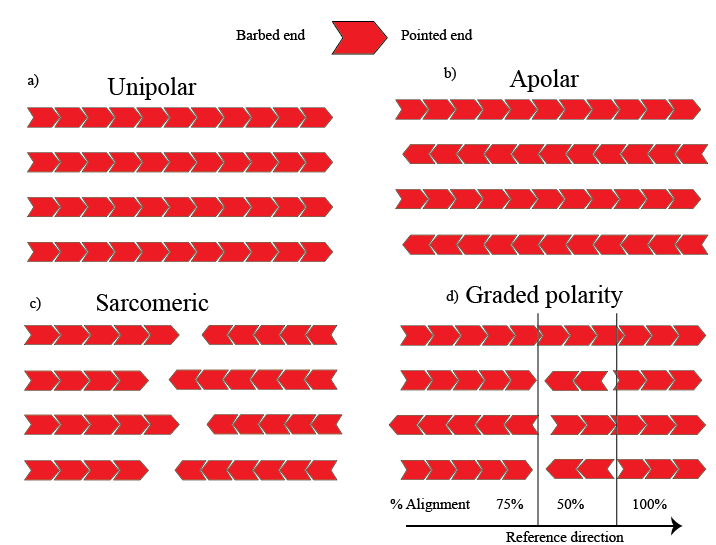


**S1 Figure. Schematic depicting different modes of actin bundle organization.**
Actin monomers with barbed and pointed end polymerize to form long filaments. a) Unipolar bundles have polarity sorted barbed and pointed ends while c) Sarcomeric bundles have polarity sorted pointed ends. b) Apolar bundles have zero net polarity while d) graded polarity bundles have varying degrees of polarity along the length of the bundle.


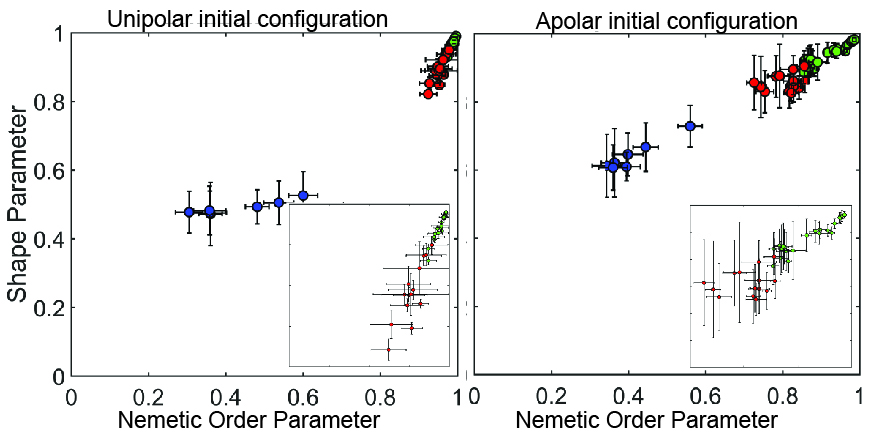


**S2 Figure Nematic order and shape parameters highlight the differences between BL, ABI and AL morphologies.**
Mean and standard deviation of nematic order parameter and shape parameter from the last 500s from networks simulated at 42 different mole ratio pairs under (a) unipolar and (b) apolar initial conditions are shown. BL, ABI and AL morphologies are colored respectively in green, red and blue respectively. Inset shows zoomed in plots of shape and order parameters to highlight differences between BL and ABI morphologies. Please refer to Supporting Material (sections 2.4.2 and 2.4.3) for definition of order and shape parameters.


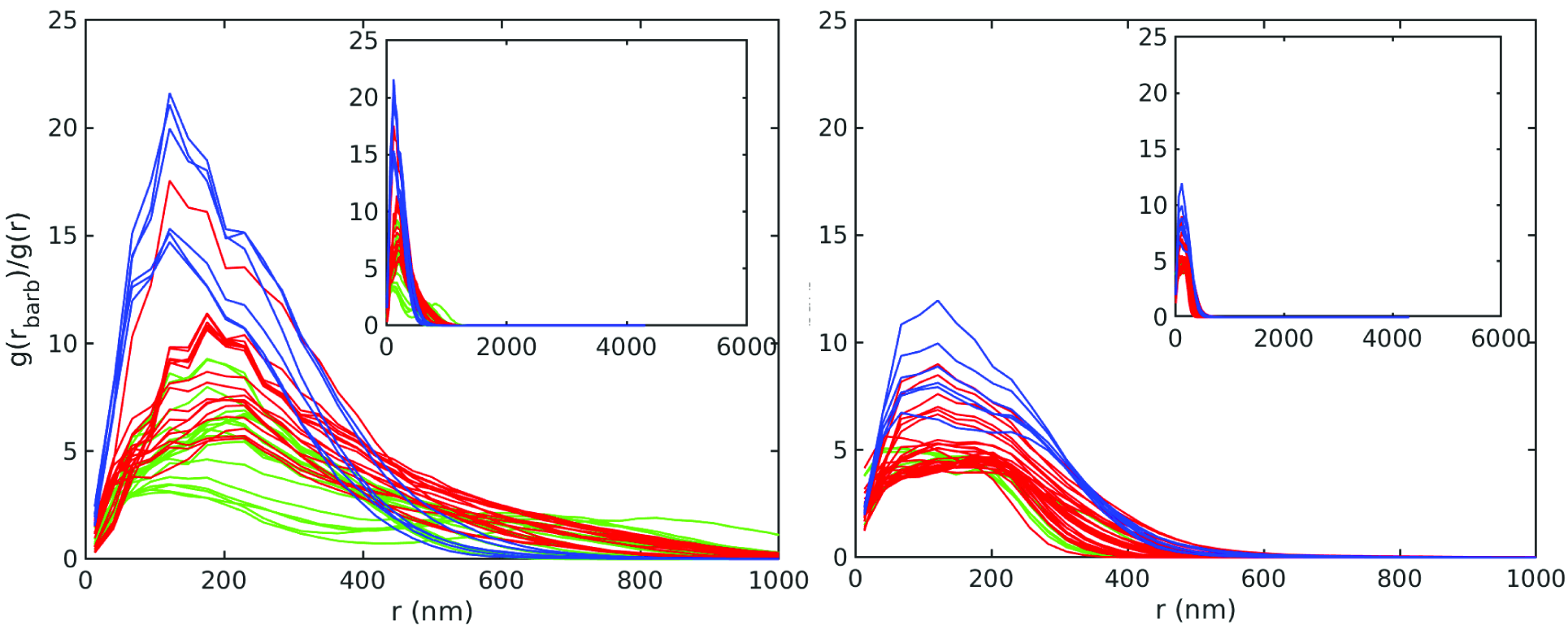


**S3 Figure Radial distribution function based order parameter for different network morphologies observed in non-treadmilling networks.** Profiles of $g(r_{barb})/g(r)$ for 42 different mole ratio (M:A,α:A) pairs are plotted for unipolar (left) and apolar (right). In studies by Freedman et al [1], this order parameter delineates polarity sorted networks. BL, ABI and AL morphologies are colored respectively in green, red and blue respectively. Inset shows complete profile of distribution function. Using the last 500s of trajectories, each actin segment was interpolated to get fine grain network structure.


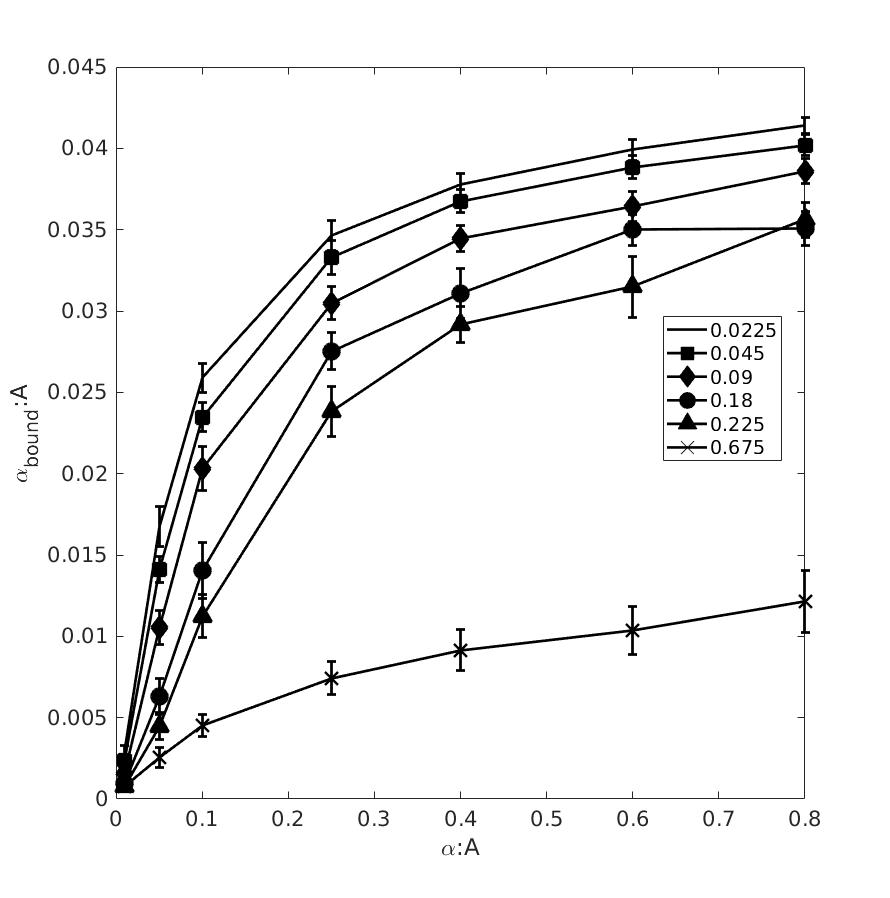

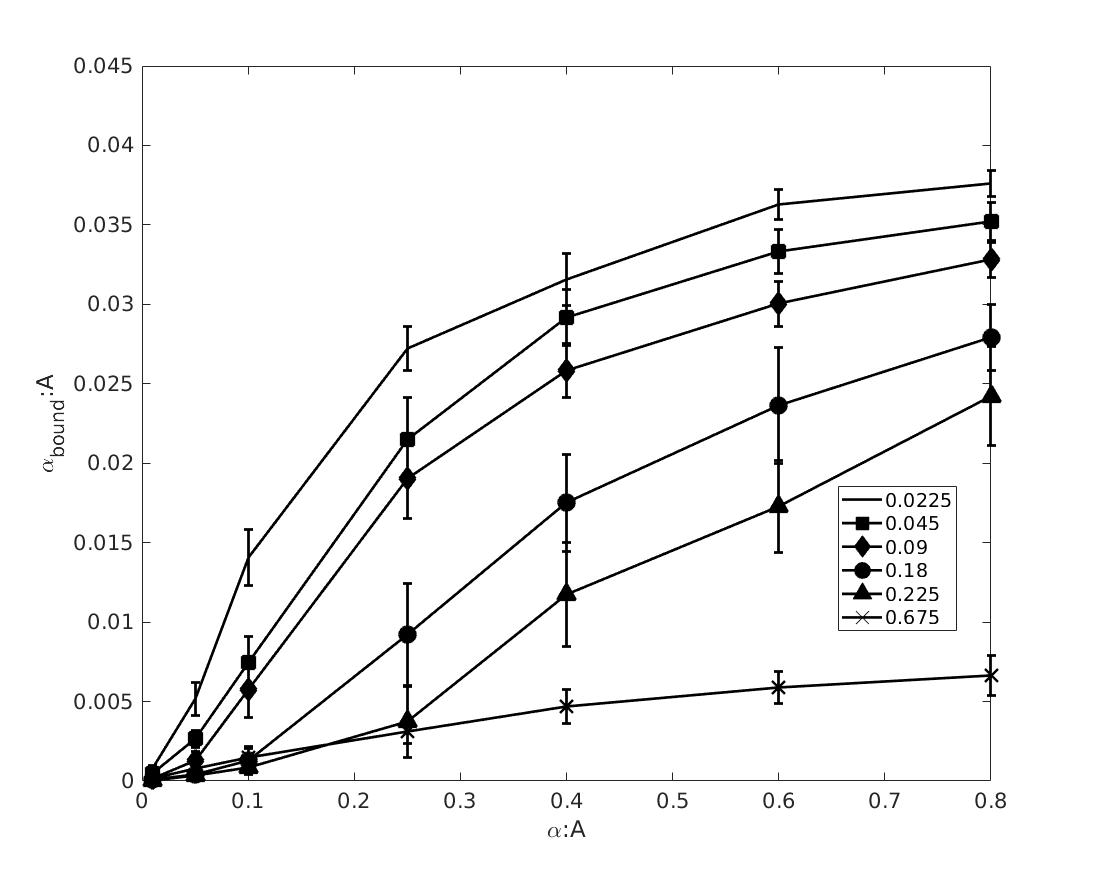
**S4 Figure. Mole ratio of crosslinkers bound to actin network at different total crosslinker mole ratios.** Mean and standard deviation bound crosslinker mole ratios (α_bound_:A) for networks evolved from both unipolar (left) and apolar (right) bundle configurations at different myosin mole ratios (M:A, legend) are shown. Last 500s of the trajectories were used to calculate bound crosslinker mole ratios. As M:S is increased, the average number of linkers bound to the network reduces.


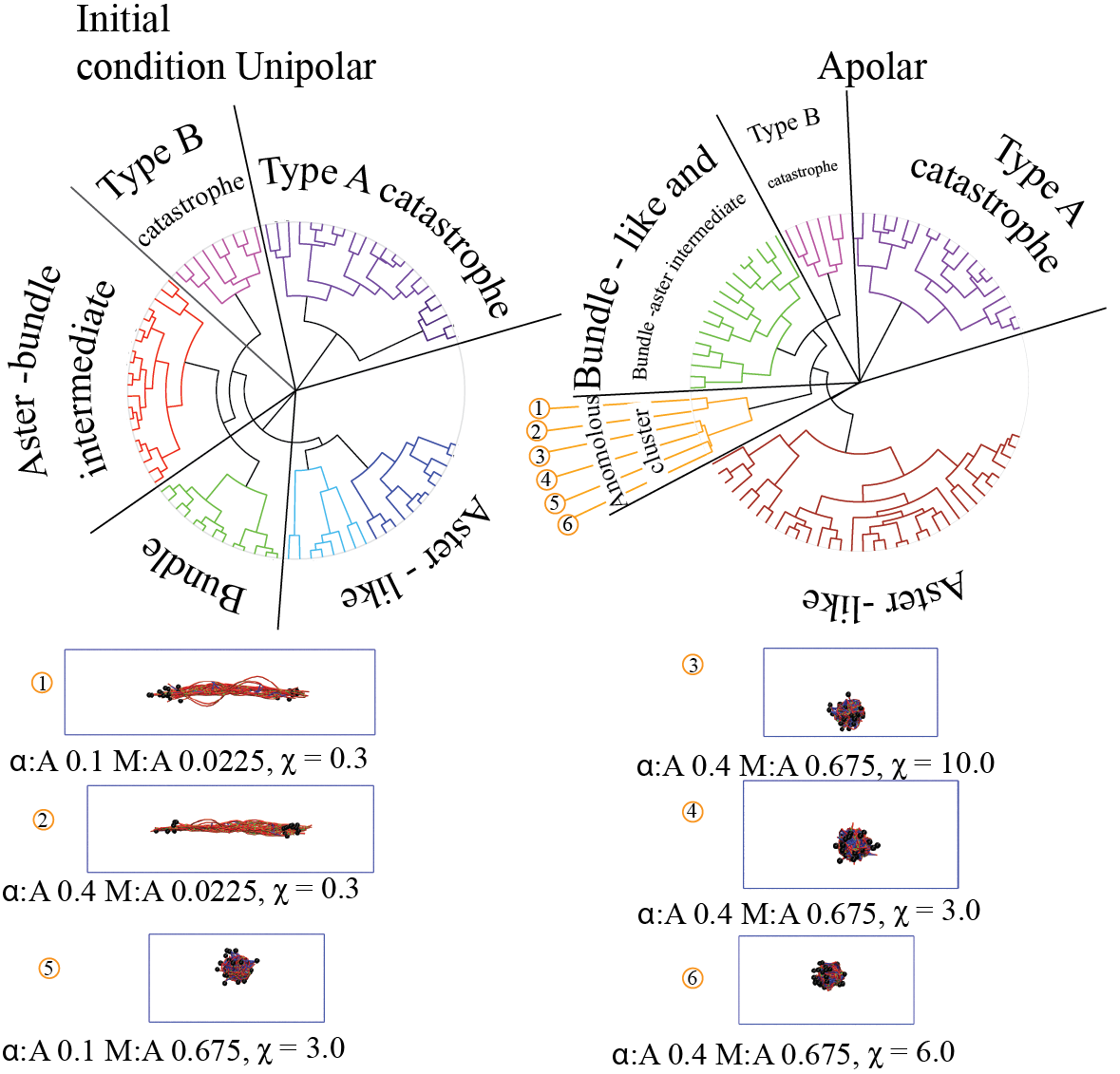

**S5 Figure. Dendrograms from clustering actin networks from unipolar (left) and apolar (right) initial conditions.** Dendrogram clades are colored based on network morphology. Type A catastrophe is characterized by a poorly connected network with very low packing density while B catastrophe is characterized by a connected network with moderate packing density. 6 of the resulting clusters from apolar conditions were incorrectly clustered to an anomalous cluster. They contained spherical asters and apolar bundles in the same clade. Representative snapshots of members within the cluster are also shown. The two kinds of catastrophes seen are characterized either by network split (Type A) of poor inter-filament connectivity (Type B). In addition, we also looked at the

**
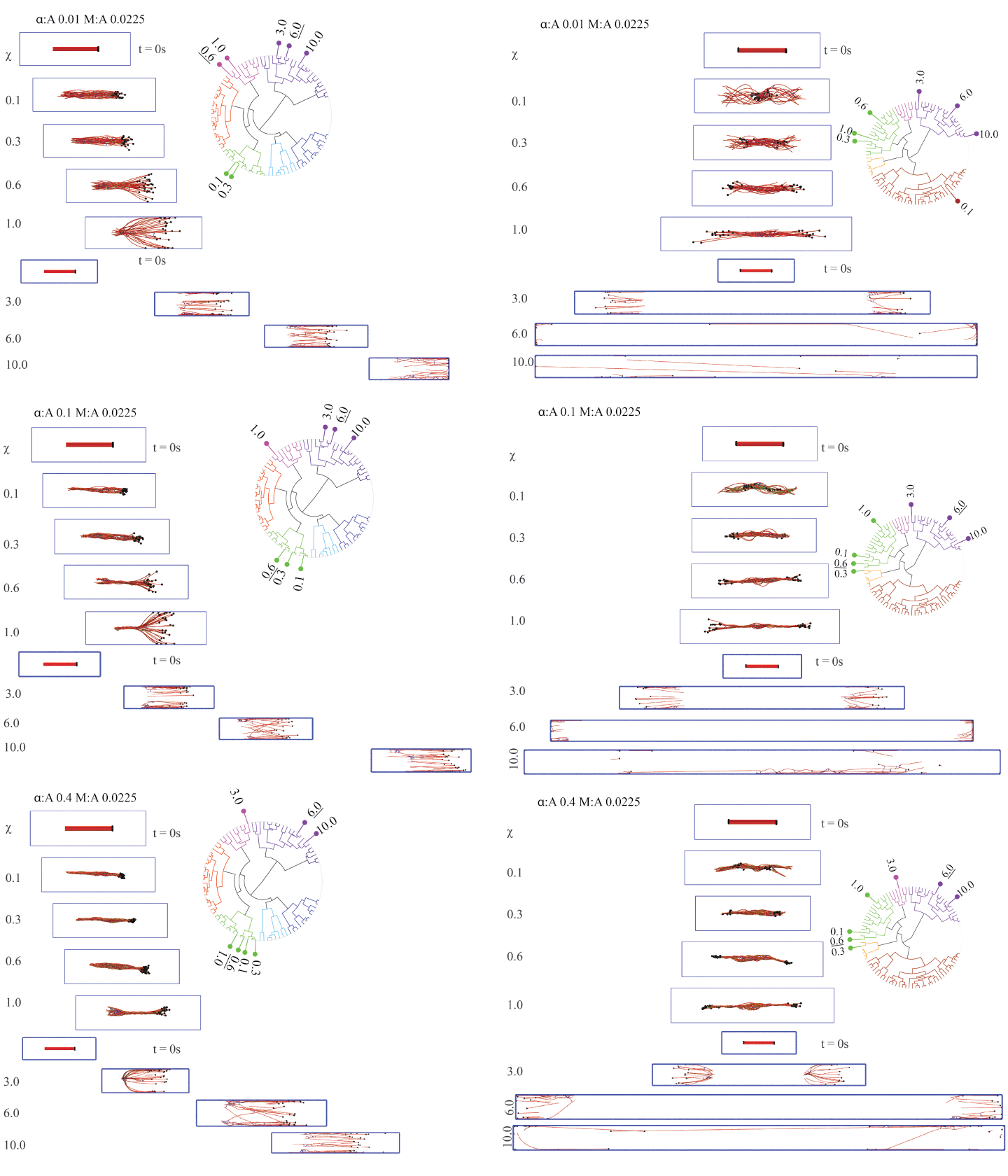

S6 Figure. Networks evolved from unipolar (left) and apolar (right) bundle configuration with M:A = 0.0225, at α:A 0.01 (top row), 0.1 (middle row), and 0.4 (bottom row) under different treadmilling rates modulated through χ.** Side view of reaction volumes (blue rectangle) filled with actin filaments (red) along with bound myosin (blue) and α-actinin (green) are shown. Initial reaction volume side views along with initial actin network configuration are also shown for comparison. Inset in each panel shows a dendrogram obtained from clustering protocol used. Refer Fig S4 for color map of dendrograms presented.

**
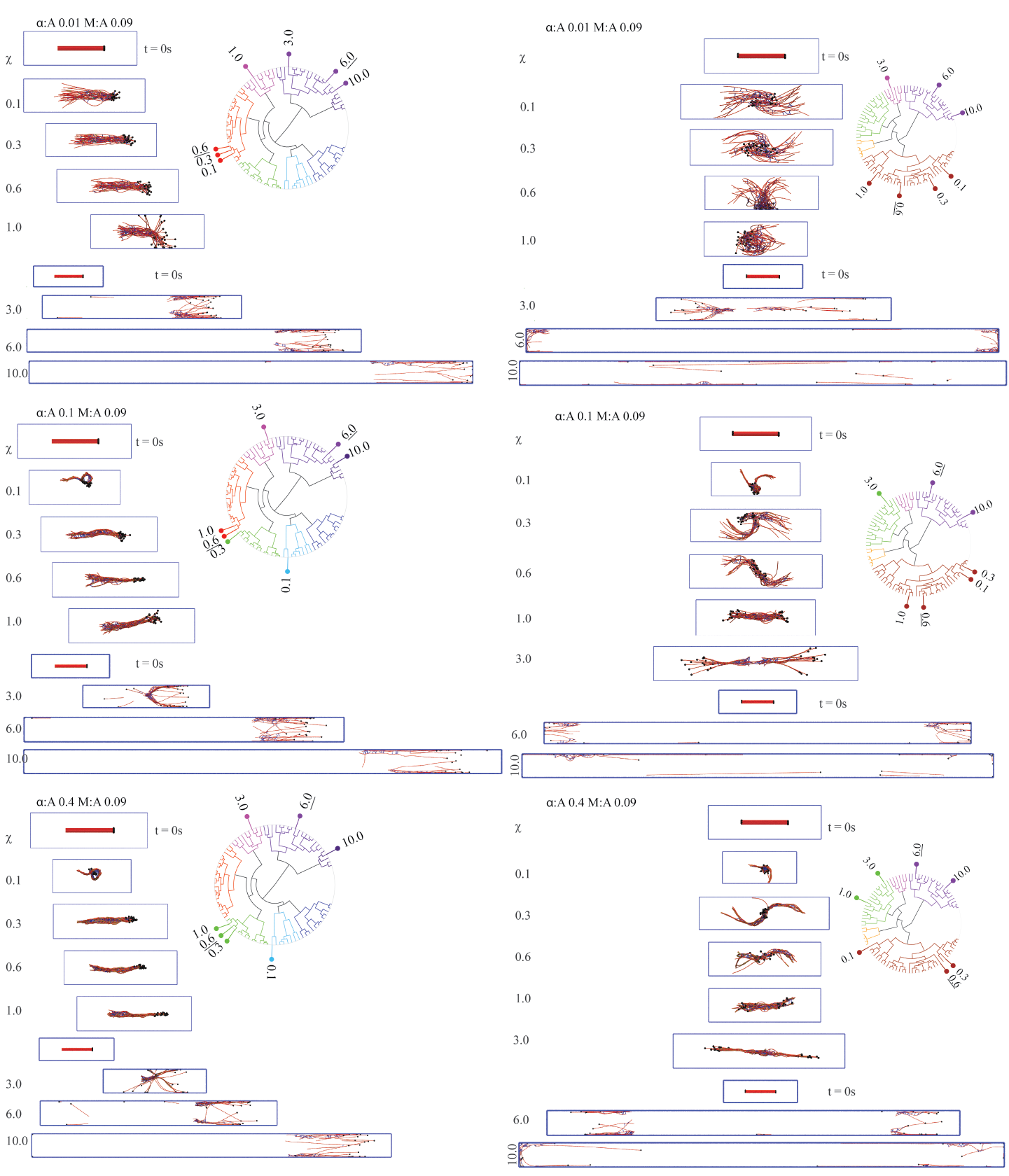

S7 Figure. Networks evolved from unipolar (left) and apolar (right) bundle configuration with M:A = 0.09, at α:A 0.01 (top row), 0.1 (middle row), and 0.4 (bottom row) under different treadmilling rates modulated through χ.** Side view of reaction volumes (blue rectangle) filled with actin filaments (red) along with bound myosin (blue) and α-actinin (green) are shown. Initial reaction volume side views along with initial actin network configuration are also shown for comparison. Inset in each panel shows a dendrogram obtained from clustering protocol used. Refer Fig S4 for color map of dendrograms presented.

**
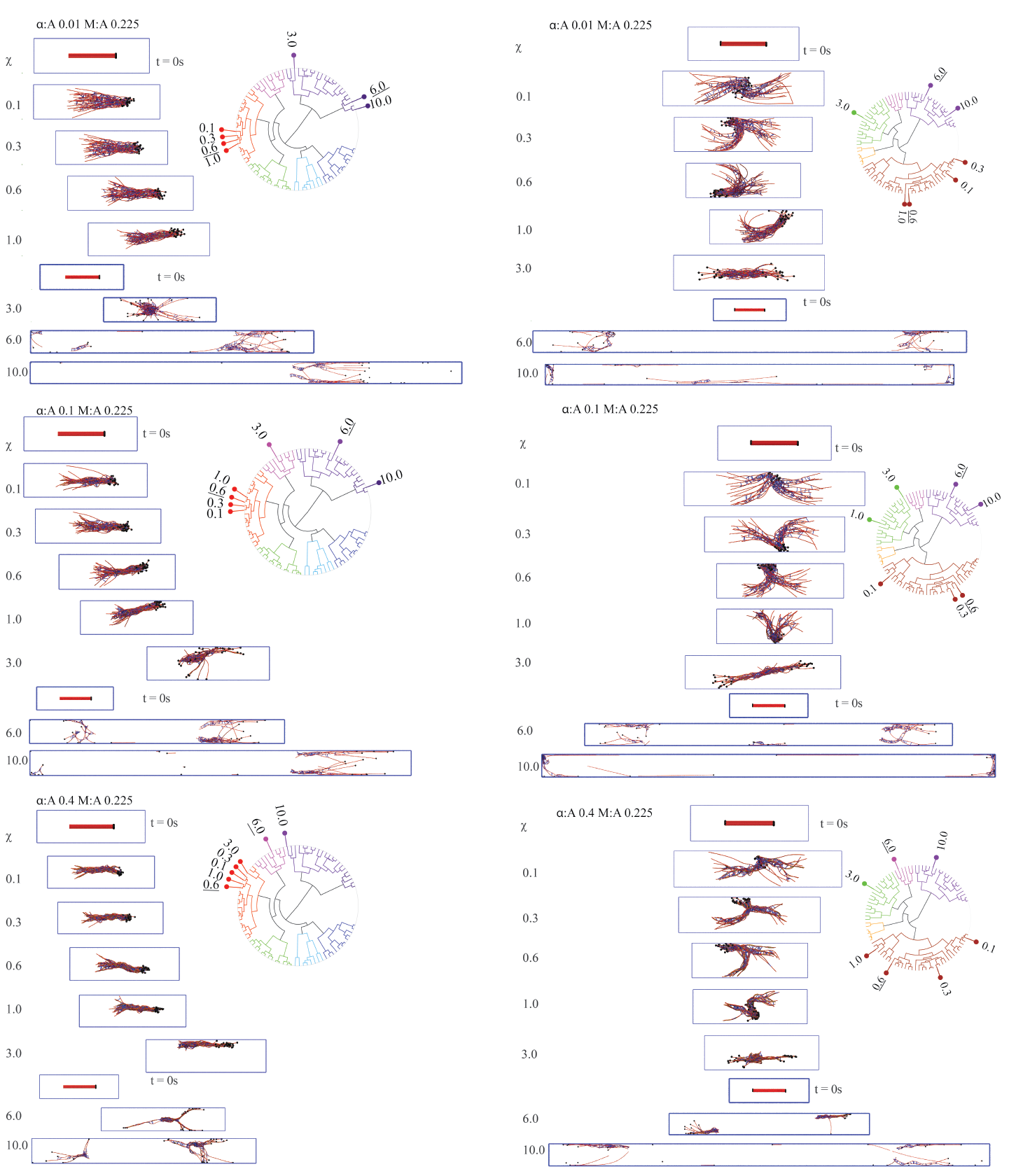
**

**S8 Figure. Networks evolved from unipolar (left) and apolar (right) bundle configuration with M:A = 0.0225, at α:A 0.01 (top row), 0.1 (middle row), and 0.4 (bottom row) under different treadmilling rates modulated through χ.** Side view of reaction volumes (blue rectangle) filled with actin filaments (red) along with bound myosin (blue) and α-actinin (green) are shown. Initial reaction volume side views along with initial actin network configuration are also shown for comparison. Inset in each panel shows a dendrogram obtained from clustering protocol used. Refer Fig S4 for color map of dendrograms presented.

**
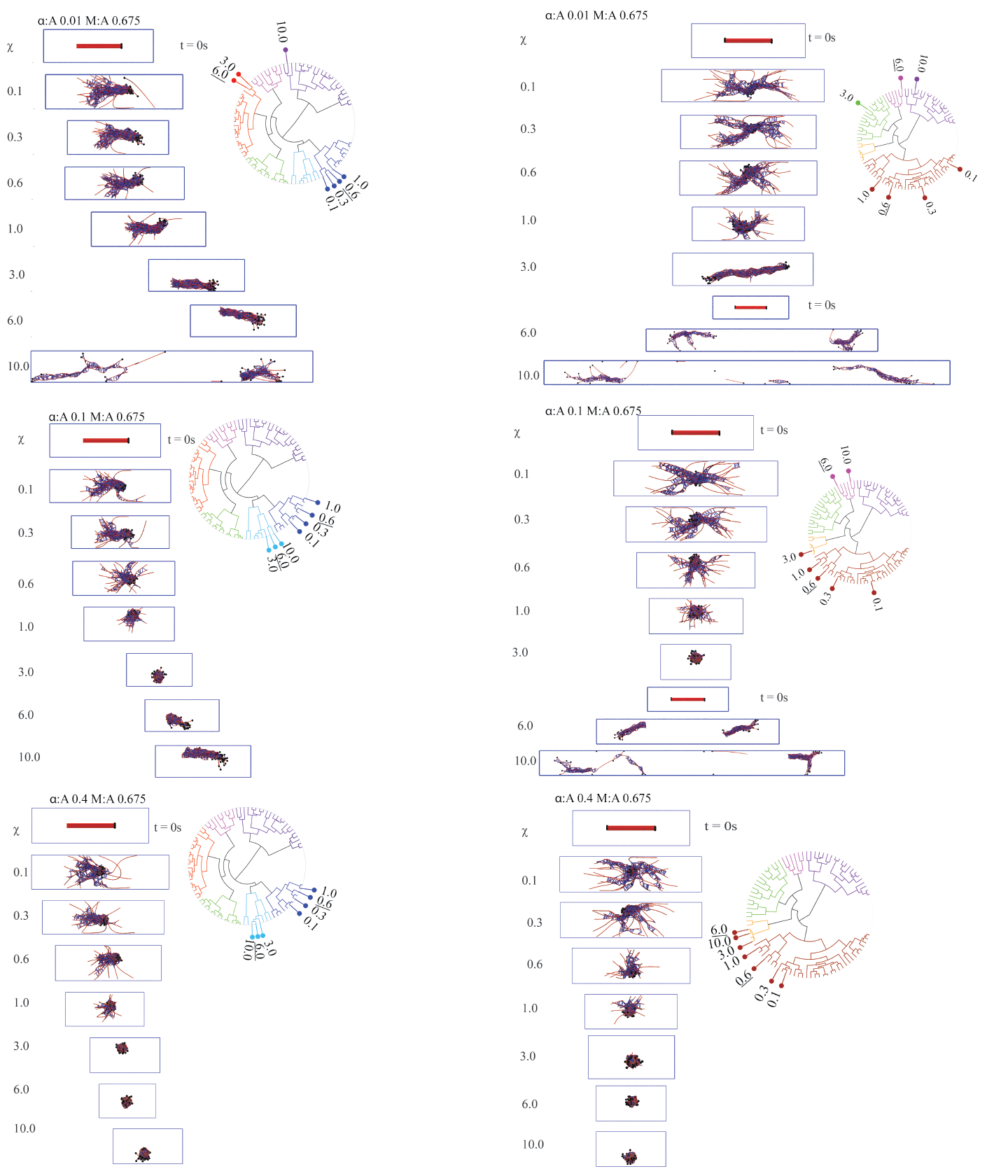

S9 Figure. Networks evolved from unipolar (left) and apolar (right) bundle configuration with M:A = 0.675, at α:A 0.01 (top row), 0.1 (middle row), and 0.4 (bottom row) under different treadmilling rates modulated through χ.** Side view of reaction volumes (blue rectangle) filled with actin filaments (red) along with bound myosin (blue) and α-actinin (green) are shown. Initial reaction volume side views along with initial actin network configuration are also shown for comparison. Inset in each panel shows a dendrogram obtained from clustering protocol used. Refer Fig S4 for color map of dendrograms presented.

# **2. Supporting Methods**

## **2.1 Chemical model**

### **2.1.1 Diffusion**

Rigorous consideration of the diffusion process would involve Brownian dynamics simulation of each of the freely diffusing molecules in the system. As this is computationally expensive, we divide the reaction volume into compartments of uniform mixing based on the Kuromoto length [2]. Kuromoto length is the length scale over which two regions of the reactive diffusion system are uncorrelated with one another with respect to copy number fluctuations of diffusing molecules [3,4].

Kuromoto length measures the mean free path before reactive collisions. In other words, a chemical species diffusing in a volume undergo reaction before it traverses Kuromoto length [5]. Within spatial dimensions determined by Kuromoto length, the species are mixed uniformly, while points separated by distances larger than Kuramoto length fluctuate independently [3,4]. Hence, the reaction volume is divided into compartments whose dimensions are smaller than Kuromoto length. If the species of interest diffusing with diffusion constant D, is produced at the mesoscopic rate k+ and consumed at the rate k-, Kuramoto length is given by the ratio

$K_{l}=\sqrt{\frac{k^{+}k^{-}}{D}}$

As actin polymerization is the fastest reaction in actomyosin networks, cellular concentration of actin is used to determine Kuromoto length. We choose a compartment size of 500 nm which is less than the Kuromoto length [6].

Diffusion reactions are defined as events of single molecule transfer between two compartments. Hence, for two compartments (A and B) sharing a boundary, we define two separate diffusion reactions (molecule from A diffuses into B and vice versa). Diffusion events are chemical reactions in the stochastic simulation protocol. Thus, diffusing molecules are represented as a homogenous continuum within compartments instead of individual particles. Diffusion coefficients of α-actinin and myosin were considered to be 1/4 and 1/8 that of actin respectively.

### **2.1.2 α-actinin, myosin minifilament binding and unbinding**

The rate constants for each of these reactions were obtained from experiments as explained in [6]. Any two unoccupied actin binding sites within (4 per cylinder) α-actinin (30-40 nm)/minifilament (175-225 nm) binding distance can be occupied by a crosslinker/NMII-A minifilament respectively. Binding site is chosen at random from the set of all possible binding sites. NMIIA minifilaments are made of 15-30 myosin molecules [7] and the number of myosin heads is chosen at random within the specified range during each binding event. Refer to the section on Mechanical Model, Mechanichemical coupling for more details.

### **2.1.3 Minifilament walking**

Minifilaments make use of energy from ATP hydrolysis and processively walk towards barbed ends of actin network. We simulate minifilament walking based on zero load walking rates used in previous studies [8]. Mechanochemical coupling of walking rates is explained later.

### **2.2 Stochastic simulation of actomyosin reaction-diffusion network.**

The most common method to represent a reaction network such as one described above is by a series of differential equations coupled with one another based on continuously varying concentrations of reacting species. However, this approach fails in biologically relevant cases as the concentration of reacting species is very low. Hence, we resort to a stochastic framework based on number of each species to represent N reactions based on mesoscopic reaction constants ($c_{\mu}$, μ $\epsilon$ [1,N]). Both stochastic and deterministic methods converge at the thermodynamic limit [9]. The set of all copy numbers of each reacting species ({X_j_}, j $\epsilon$ [1,M]) represents the instantaneous state of such a network of dimension M equal to the number of reacting species. One of the ways of solving such a network is to write a master equation [10,11]. Such methods often tend to be analytically intractable. Hence, Gillespie proposed a method [12] through which trajectories are obtained by picking the time and the reaction at random in each step based on a propensity (a_μ_). The propensity is defined as,

$a_{\mu}=\gamma_{\mu}c_{\mu}$ (S1)

where $\gamma_{\mu}$ is the degeneracy in the number of ways the reaction could be executed. For example, in the case of $2A\to B$ with $X_{A}$ copies of *A*, $\gamma={X_{A}\left( X_{A}-1 \right)}/2$. Mesoscopic rate constant $c_{\mu}$ can be obtained from deterministic rate constant k_μ_ corresponding to a reaction of order *n* in a reaction volume *V_r_* by,

$c_{\mu}=k_{\mu}\left( \frac{V_{r}}{N_{A}} \right)^{n-1}$ (S2)

where *N_A_* represents Avagadro number. In this study, we use *next reaction method* to simulate actomyosin reaction network.

### **2.2.2 Next reaction method**

The *Next reaction method* [13] based on the *first reaction method* involves the following steps*.* Initially, both the time step $\tau_{\mu}$ ($\tau_{\mu}=\left( 1/{a_{\mu}} \right)ln\left( 1/{r_{\mu}} \right)$) and propensity $a_{\mu}$ are calculated for each reaction where $r_{\mu}$ is a random number between 0 and 1. The reaction R with the smallest $\tau_{\mu}$ is executed and the {$a_{\mu}$} that depend on R alone are updated. This is made possible by use of a dependency graph for the reaction network. Then, the reactions are efficiently sorted based on their $\tau_{\mu}$ using an indexed priority queue data structure. This algorithm offers great reduction in computational complexity for simulation of a reaction network. This method is used to simulate unipolar actomyosin bundles in this study.

## **2.2. Details of mechanical model in MEDYAN**

### **2.2.1. Actin filaments**

In MEDYAN, actin filaments are modeled as cylinders with equilibrium spacing $l_{0}^{m}\ll l_{p}$ ($l_{0}^{m}$= 108 nm in this study, $l_{p}$-persistence length) connected in the ends. This aids considerable speed up during mechanical equilibration of the network in comparison with traditional bead and spring models. Axial stretching ($U_{i}^{str}$), bending at the hinges between consecutive cylinders ($U_{i}^{bend}$), excluded volume interactions between cylinders are the potentials included in the model.

The stretching and bending potentials are given by,

$U_{i}^{str}=\frac{1}{2}K_{str}\left( \left| \vec{l_{i}} \right|-l_{0} \right)^{2}$ (S3)

$U_{i}^{bend}=\varepsilon_{bend}\left( 1-cos\left( \theta_{i,i+1} \right) \right)$ (S4)

$K_{str},\varepsilon_{bend}$ are stretching and bending constants respectively. $\left| \vec{l_{i}} \right|$ is the length of cylinder *i* connecting beads at $\vec{x_{i}^{1}}$ and $\vec{x_{i}^{2}}$. $\theta_{i,i+1}$ measures the angle between cylinders *i* and *i+1.* Please refer to Fig 2 for an illustration on potentials used in the model.

In the case of excluded volume effects, we consider a potential equal to $\left| \vec{r_{i}}-\vec{r_{j}} \right|^{4}$ between any two fragments on cylinders *i* and *j* located at $\vec{r_{i}}$ and $\vec{r_{j}}$ respectively. Thus,

$U_{ij}^{vol}=K_{vol}\int_{l_{i}} \int_{l_{j}} \frac{dl_{i}dl_{j}}{\left| \vec{r_{i}}-\vec{r_{j}} \right|^{4}}$ (S5)

Where $K_{vol}$ determines strength of repulsion. This can be rewritten by considering $\vec{r_{i}}=\vec{x_{i}^{1}}+t\left( \vec{x_{i}^{2}}-\vec{x_{i}^{1}} \right)$, where *t* is a parameter.

$U_{ij}^{vol}=K_{vol}\int_{0}^{1} \int_{0}^{1} \frac{dsdt}{\left| \vec{r_{i}}-\vec{r_{j}} \right|^{4}}$ (S6)

In this stuy, we consider 4 binding sites per cylinder available exclusively for linker, motor binding.

### **2.2.2. α-actinin, minifilament model**

As soon as a binding event is triggered, the actin binding protein is represented physically as a spring connecting a pair of binding sites (on actin filament) subject to experimental constraints of bond length. During unbinding, the actin bound protein is replaced back in the reaction volume. Minifilaments can be made of 15 to 30 heads and a number is chosen at random during each binding step. Crosslinkers and minifilaments bound to points *i* and *j* on actin filament experience stretching potentials given by,

$U_{ij}^{MF}=\frac{1}{2}K_{str}^{MF}\left( \left| \vec{l_{ij}} \right|-l_{0} \right)^{2}$ (S7)

$U_{ij}^{linker}=\frac{1}{2}K_{str}^{linker}\left( \left| \vec{l_{ij}} \right|-l_{0} \right)^{2}$ (S8)

In the case of minifilaments (MF), $\vec{l_{ij}}$ changes with motor walking. It is rewritten in terms of fractional position of the two MF heads on the actin cylinder.

### **2.2.2. Boundary**

Boundaries in MEDYAN repel cylinders to confine the network within a certain volume. The repulsion energy $\varepsilon_{boundary}$ scales exponentially with characteristic length $\lambda$.

$U_{i}^{boundary}= \varepsilon_{boundary}e^{-d/\lambda}$ (S9)

## **2.3 Mechanochemical coupling**

MEDYAN framework allows for explicit consideration of feedbacks from mechanics to the rates of chemical events. Anisotropy in actin network leads to spatial differences in loads acting on actin, minifilaments and α-actinin molecules. This highlights the necessity of mechanochemical coupling as experimental evidence suggests that minifilaments in regions of high stress undergo dynamics in accordance with catch-slip model while the α-actinin molecules behave in accordance with a slip model.

### **2.3.1. Minifilament walking, binding and unbinding rates**

The salient features of minifilaments of non-processive myosin II protein are well explained by parallel cluster model (PCM) [8]. Ideas of mechanochemical feedback are borrowed primarily from this model. We consider a two state system for minifilaments namely bound and unbound. Bound state configuration is characterized by AM.ADP (Actin bound myosin with ADP ligand) while unbound state involves M.ADP.Pi (freely diffusing minifilament liganded to ADP and Pi). Binding, unbinding and walking rates are affected by mechanochemical feedback.

The filament binding rate $k_{b}$ of minifilament can be written as,

$k_{fil,bind}=k_{NMIIA,bind}\cdot N_{total}$ (S10)

where $k_{NMIIA,bind}$is the binding rate for a single myosin head. $N_{total}$ is picked at random at every binding event within the minimum and maximum number of heads specified.

When subject to an external load ($F_{ext}$), the unbinding and walking rates are affected based on number of heads in bound state ($N_{bound}$). Under zero load conditions ($F_{ext}=0$), $N_{bound}$ is given by ${N_{0}=\rho N}_{total}$. ρ=0.1 [14] is duty ratio defined as the fraction of time a myosin head stays in strongly bound state. Under non -zero $F_{ext}$, we assume linear scaling of $N_{bound}$ with parameter γ=0.05.

$N_{bound}=N_{0}+\gamma F_{ext}$ (S11)

We use a catch bond behavior similar to that of [15] as the forces in our system are within slip bond threshold [6].

$k_{fil,unbind}=\frac{\epsilon\cdot k_{NMIIA,unbind}^{0}}{N_{bound}}\cdot exp\left( \frac{-F_{ext}}{N_{bound}\cdot F_{NMIIA,unbind}} \right)$ (S12)

where $F_{NMIIA,unbind}$ is the characteristic unbinding force of each myosin head. *ϵ* is chosen to be 0.2 /pN.

Under zero load conditions, walking rates are of each minifilament (not for individual heads) is related to single myosin head binding rate as,

$k_{walk}^{0}= {k_{NMII,bind}\left( N_{total}-N_{bound}^{0} \right)}/{N_{bound}^{0}}$ (S13)

In the case of walking rate, we employ a Hill [16] type force-velocity relation to account for mechanochemical feedback with a parameter $\zeta$=0.1 [8].

$k_{walk}=k_{walk}^{0}\cdot\frac{\left( F_{stall}-F_{\mathrm{ext}} \right)}{\left( F_{stall}+{F_{ext}}/\zeta\right)}$ (S14)

where $F_{stall}=12.6pN$ is the minifilament stall force and $\zeta=0.1$.

### **2.3.2 α-actinin unbinding rate**

Experimental evidence suggests that α-actinin binding rate is unaffected by external load while the unbinding rate depends as a slip bond behavior.

$k_{\alpha,unbind}=k_{\alpha,unbind}^{0}\cdot exp\left( \frac{F_{ext}}{F_{\alpha,unbind}} \right)$ (S15)

## **2.4 Trajectory analysis**

Trajectories were generated for each pair of crosslinker and myosin mole ratios considered (α:A, M:A) in the case of non-treadmilling study, and for each triad of treadmilling factor, crosslinker mole ratio and myosin mole ratio (χ, α:A, M:A) to study effect of treadmilling under both unipolar and apolar initial conditions. Trajectories are analyzed once steady state is reached. Steady state is defined based on network radius of gyration for non-treadmilling networks (see Fig S10) and based on filament length fluctuations for treadmilling networks (see Fig S11). Filament length fluctuations are characterized by filament treadmilling rate defined as the rate of monomer addition at plus end or removal at minus end of each filament in the network. In order to ensure equal sampling at steady state across all conditions studied, last 500s of the trajectories are considered for analyses.


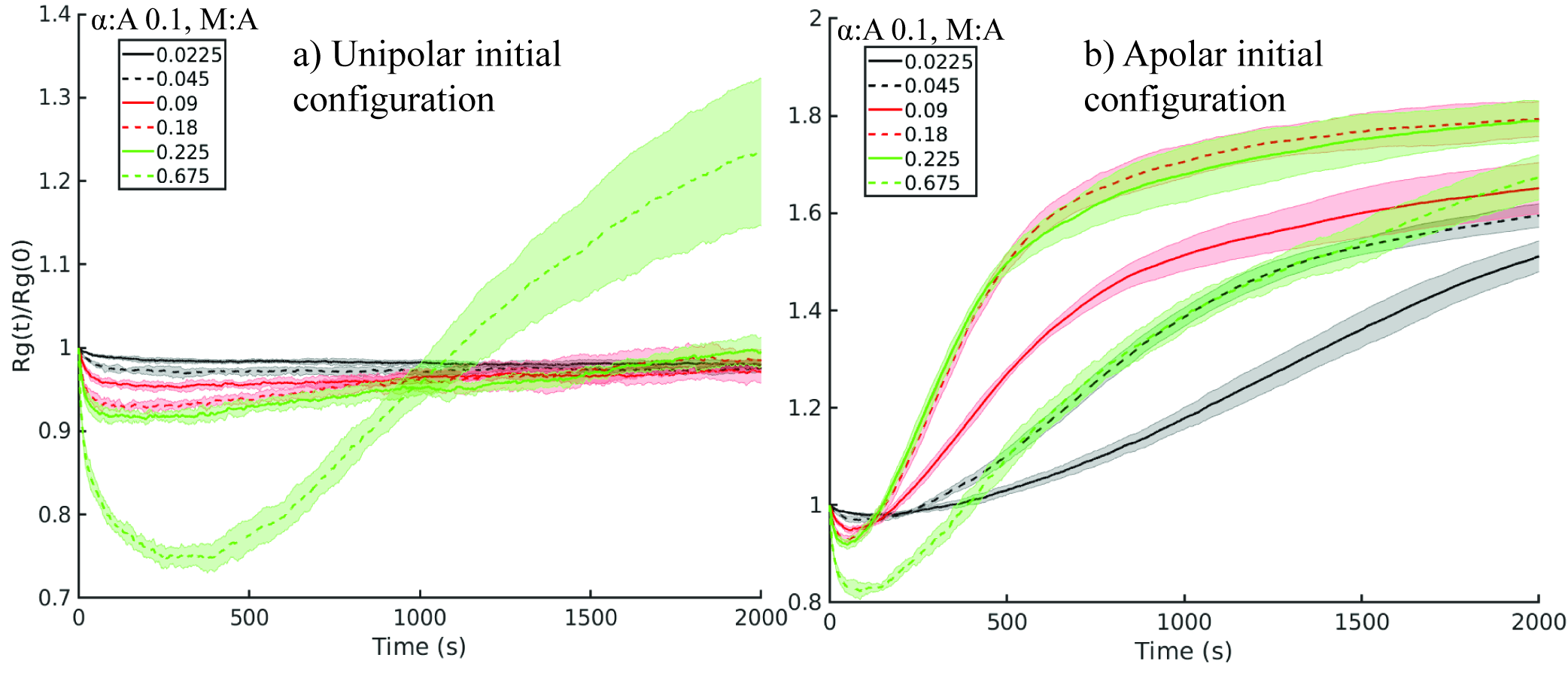

**S10 Figure. Ratio of network radius of gyration to initial radius of gyration at α:A 0.1 and various myosin mole ratios.** Mean and standard deviation of Rg(t)/Rg(0) is plotted as a time series for trajectories from a) Unipolar b) Apolar initial configurations. Both unipolar and apolar bundles at high myosin mole ratio (0.675) show higher fluctuation in Rg(t)/Rg(0) due to myosin activity.


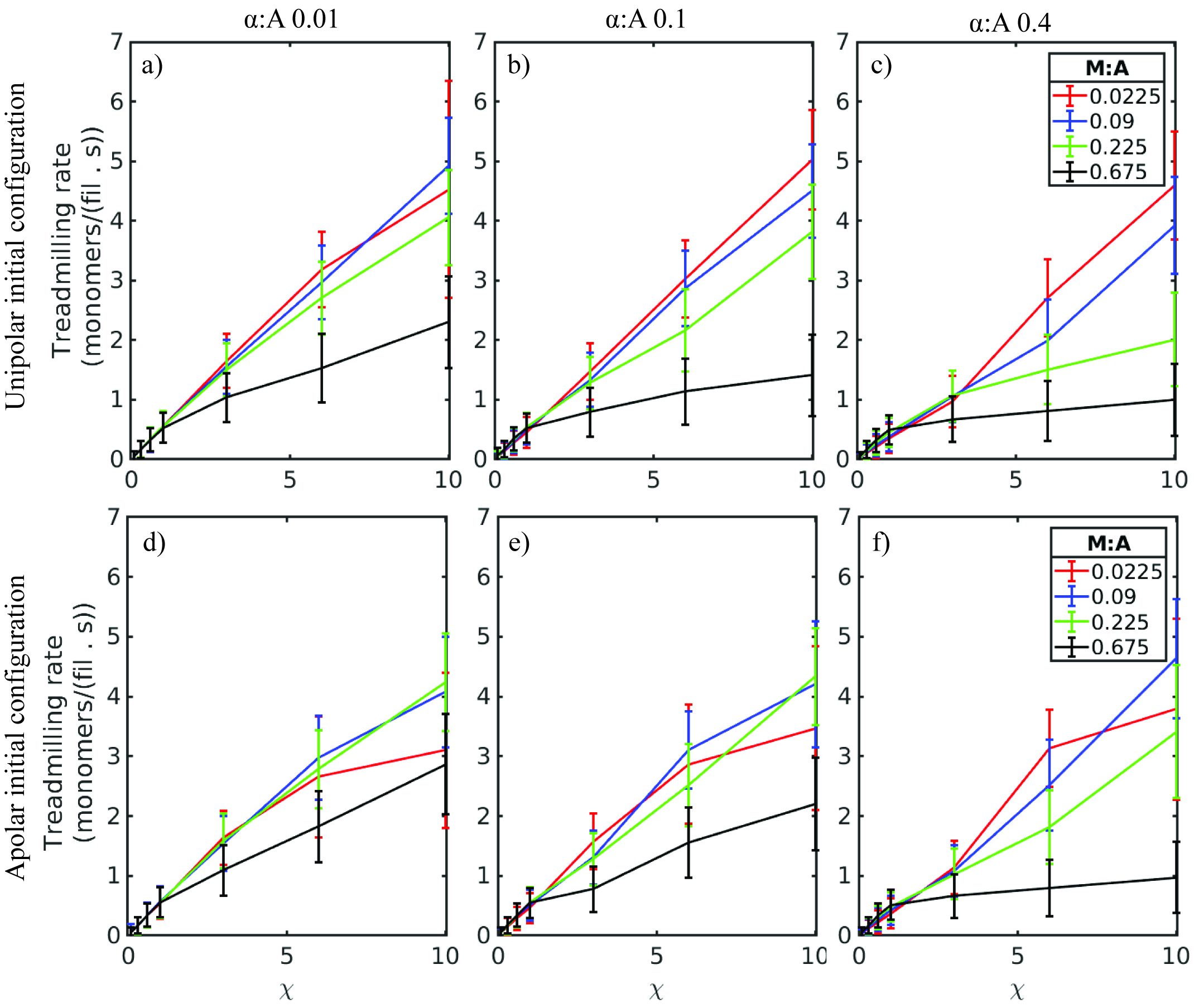


**S11 Figure. Treadmilling rates as a function of χ parameter.** Mean and standard deviation of treadmilling rates of networks evolved from unipolar (a-c) and apolar (d-f) bundles as initial configuration are shown. α-actinin mole ratios are mentioned on top of each panel at varying M:A. Last 500s of the trajectories were used to calculate treadmilling rates. Treadmilling rate is defined as the rate of monomer addition at plus end (removal at minus end) for each filament in the network.

### **2.4.1. Clustering analysis**

In order to understand underlying network morphologies resulting from varying α-actinin and myosin minifilament mole ratios under unipolar and apolar initial conditions, we devise a clustering algorithm.

**
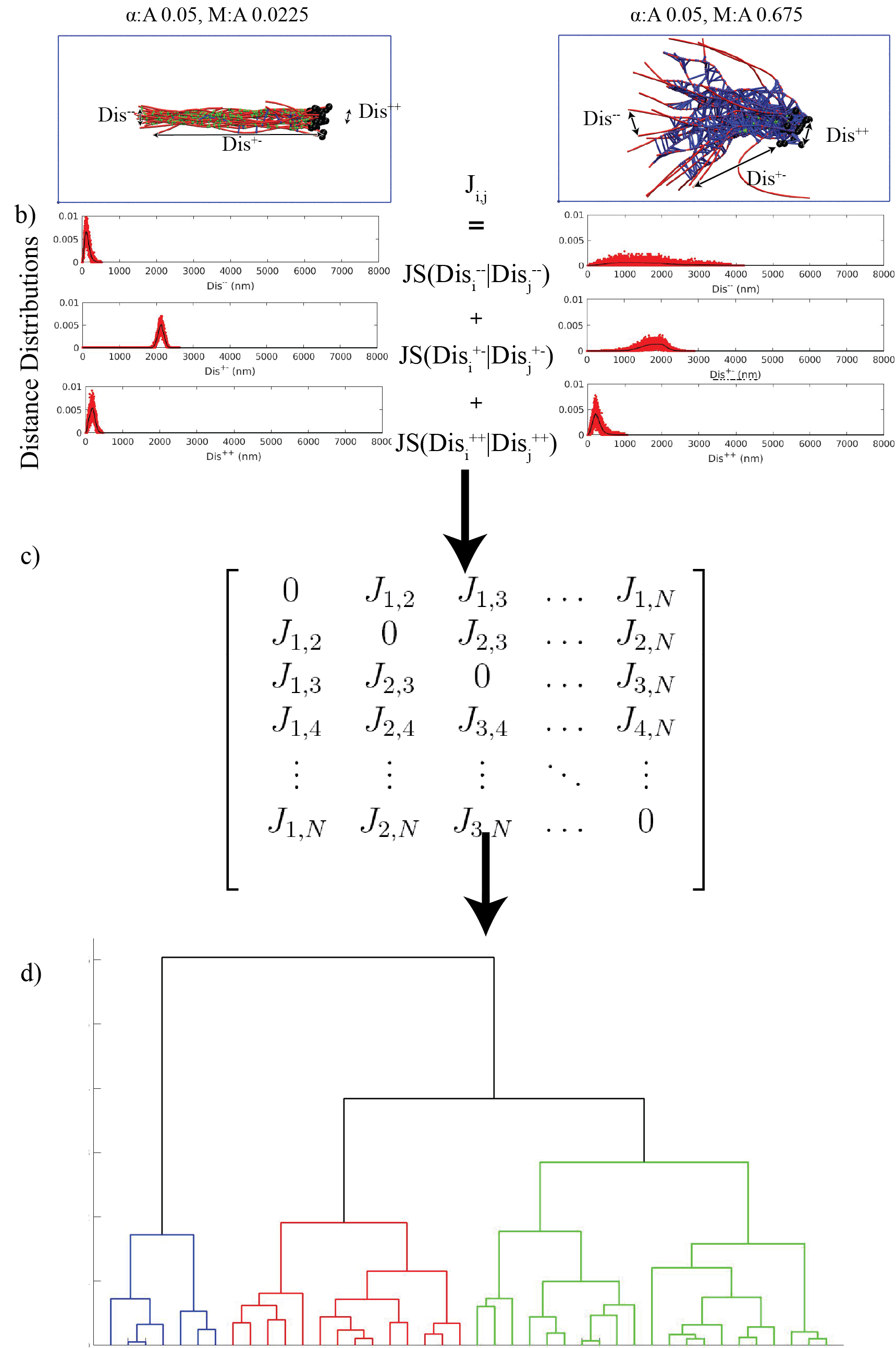
**

**S12 Figure. Schematic of clustering algorithm used to classify networks of unipolar networks under varying α:A and M:A values.** Representative final snapshots from simulations under two different M:A values, namely 0.0225 (left) and 0.675 (right) at α:A 0.01 is shown. b) Three pair-wise distance distributions corresponding to Dis^++^, Dis^—^ and Dis^+-^ are constructed. Jensen Shannon divergences of Dis^++^, Dis^—^ and Dis^+-^ are calculated for each unique simulation condition (determined by α:A and M:A) taken pairwise c) The sum of divergences is used to construct two disssmilarity matrices corresponding to unipolar and apolar initial conditions. d) Complete-linkage clustering algorithm is employed to construct an agglomerative, hierarchical linkage which can be visualized as dendrograms.

Distances between plus ends (Dis^++^), minus ends (Dis^--^) and minus-plus ends (Dis^+-^) from the last 500s of each unique simulation condition are converted to distributions (Fig S10b). To compute dissimilarity between each pair of conditions (for ex, α:A 0.05, M:A 0.0225 vs α:A 0.05, M:A 0.675), the Jensen Shannon divergence [17] measure for each of the three distributions is added to produce a dissimilarity matrix (Fig S10c). Jensen Shannon divergence between two continuous distributions P and Q is given by,

$JSD(P|\left| Q \right)=\frac{1}{2}D(P|\left| M \right)+ \frac{1}{2}D(Q||M)$ (S16)

Where M=(P+Q)/2 and D() refers to Kullback-Leibler divergence defined as,

$D_{KL}(P|\left| Q \right)= \int_{-\infty}^{\infty} p\left( x \right)log\left( \frac{p(x)}{q(x)} \right)$ (S17)

Where p and q are probability densities of P and Q.

Hierarchical agglomerative clustering (complete-linkage clustering) is employed to convert this matrix to a dendrogram (Fig S10d). The clades in the dendrograms correspond to different clusters present in our sample space. Conditions within each cluster result in similar network morphology compared to conditions outside. Similar procedure is repeated for results from varying χ-parameter to understand the relevant network morphologies.

**2.4.2. Orientational order parameter (S)** measures the directional order in liquid crystals was used to measure structure of actin bundles obtained from MEDYAN simulations. The order parameter (S) is obtained from ordering tensor $Q$ defined as,

$Q_{\alpha\beta}=\frac{3}{2}\left( \frac{1}{N}\sum_{i=0}^{N} u_{i\alpha}u_{i\beta}-\frac{1}{3}\delta_{\alpha\beta} \right) , \alpha,\beta=x,y,z$ (S18)

The largest eigenvalue obtained from matrix $Q$ represents order parameter of the snapshot. Order parameter from snapshots after t=1000s was averaged over 15 trajectories to obtain $\left\langle S \right\rangle$. Order parameter 0 corresponds to random network while 1 represents absolute alignment.

**2.4.3. Shape Parameter (Sh)** measures the ratio of variance in distribution of actin along primary axis as a function of sum of variances along all three principal axis. A matrix **R** matrix of bead coordinates is defined as,

$\boldsymbol{R}^{T}=\left[ \begin{matrix} x_{1}-\bar{x} & x_{2}-\bar{x} & \ldots x_{N}-\bar{x} \\ y_{1}-\bar{y} & y_{2}-\bar{y} & \ldots y_{N}-\bar{y} \\ z_{1}-\bar{z} & z_{2}-\bar{z} & {\ldots z}_{N}-\bar{z} \end{matrix} \right]$ (S19)

Eigen decomposition of **R^T^R** gives eigenvalues λ_1_ ≥ λ_2_ ≥ λ_3_ that correspond to variance along the three principal axes. Shape parameter is defined as,$Sh=\lambda_{1}/(\sum\lambda_{i} )$.

### **2.4.4. Probability distribution of linker, motor**

For the cases where bundle configuration is preserved and the bundle axis can be approximated by the principal axis given by eigenvector corresponding to the largest eigenvector of Eq. 11. Projections of position vectors of myosin minifilaments (α-actinin) from the center of mass of bundle gives position of minifilament (α-actinin) along the bundle principal axis. Coordinates of two binding sites and center of mass of minifilament (α-actinin) were considered. Probability density profile is obtained from binning results last 500s snapshots from all trajectories.

## **2.5 Protocol for flexible volume simulations**

At the end of each mechanical equilibration phase, we find the compartments spanned by F-actin. An additional 1 micron skin distance is added to the extremities to define the bounds along x-axis for the new reaction volume. If the new reaction volume is larger than the old reaction volume, 50% of the diffusing species in the compartments that form the bounds of old reaction volume is shared with the newly included compartments as shown in Fig S10. This is continued recursively till the compartment extremities of the new reaction volume are reached. On the other hand, if the new reaction volume is smaller, starting from the outside, all diffusing species are transferred inward along X-axis recursively to the bounds of new reaction volume.


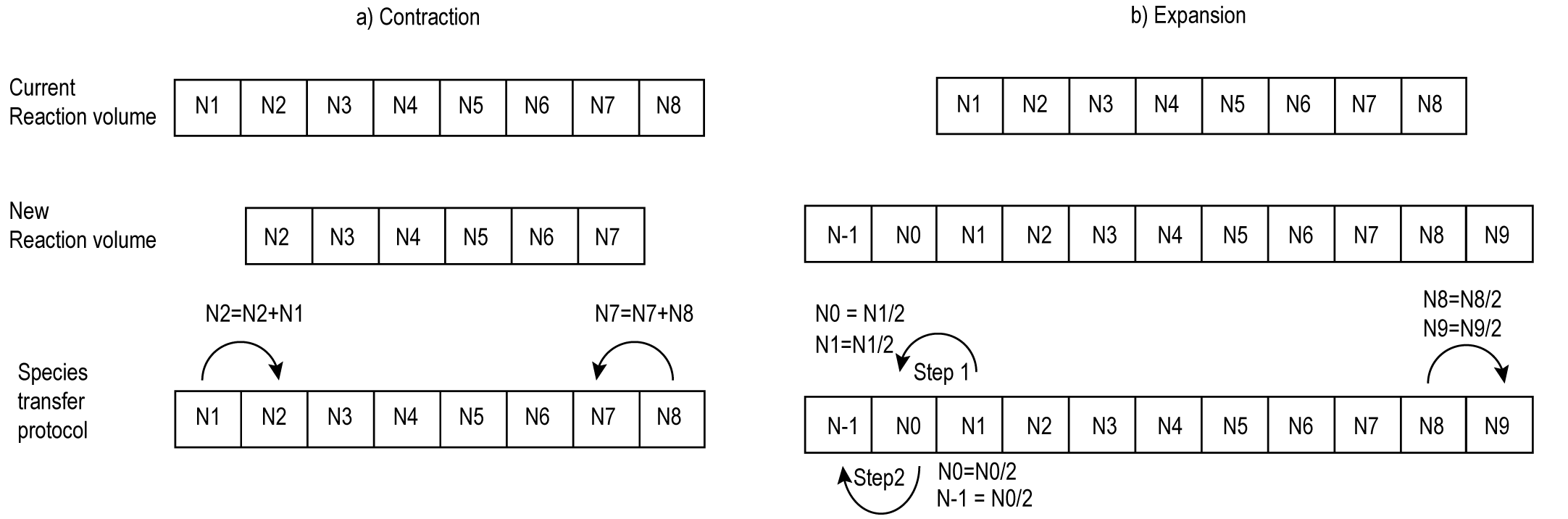


**Figure S10. Schematic explaining the flexible volume protocol employed to simulate treadmilling bundles.** Reaction volume discretized into compartments is shown. Copy number of diffusing species (N) in compartment (i) is represented as Ni. Scenarios where reaction volume has contracted (a) and expanded (b) at the end of chemical evolution and mechanical equilibration are shown. Diffusing species are redistributed to account for the change in reaction volume. This protocol helps us explore filament dynamics without the influence of boundary along the filament axis.

# **References**

1. Freedman SL, Hocky GM, Banerjee S, Dinner AR. Nonequilibrium phase diagrams for actomyosin networks. Soft Matter. Royal Society of Chemistry; 2018;14: 7740–7747. doi:10.1039/c8sm00741a

2. Kuramoto Y. Effects of Diffusion on the Fluctuations in Open Chemical Systems. Progress Theor Phys. 1974;52: 711–713. doi:10.1143/PTP.52.711

3. Kampen VNG. Fluctuations in continuous systems. AIP Conf Proc. 1976;27: 153–186. doi:10.1063/1.30357

4. Kampen VNG. The equilibrium distribution of a chemical mixture. Phys Lett A. 1976;59: 333–334. doi:10.1016/0375-9601(76)90398-4

5. Lecca P, Laurenzi I, Jordan F. Deterministic Versus Stochastic Modelling in Biochemistry and Systems Biology [Internet]. Deterministic Versus Stochastic Modelling in Biochemistry and Systems Biology. 2013. doi:10.1533/9781908818218.348

6. Popov K, Komianos J, Papoian GA. MEDYAN : Mechanochemical Simulations of Contraction and Polarity Alignment in Actomyosin Networks. PLoS Comput Biol. 2016;12: e1004877. doi:10.1371/journal.pcbi.1004877

7. Verkhovsky AB, Svitkina TM, Borisy Gary G. Myosin II filament assemblies in the active lamella of fibroblasts their morphogenesis and role in the formation of actin filament bundles. J Cell Biol. 1995;131: 989–1002.

8. Erdmann T, Albert PJ, Schwarz US. Stochastic dynamics of small ensembles of non-processive molecular motors: The parallel cluster model. J Chem Phys. 2013;139. doi:10.1063/1.4827497

9. Kurtz TG. The Relationship between Stochastic and Deterministic Models for Chemical Reactions. J Chem Phys. 1972;57: 2976. doi:10.1063/1.1678692

10. Gardiner CW. Handbook of stochastic methods. The effects of brief mindfulness intervention on acute pain experience: An examination of individual difference. 2015. doi:10.1017/CBO9781107415324.004

11. McQuarrie DA. Stochastic approach to chemical kinetics. J Appl Probab. 1967;4: 413–478. doi:10.2307/3212214

12. Gillespie DT. A general method for numerically simulating the stochastic time evolution of coupled chemical reactions. J Comput Phys. 1976;22: 403–434. doi:10.1016/0021-9991(76)90041-3

13. Gibson MA, Bruck J. Efficient Exact Stochastic Simulation of Chemical Systems with Many Species and Many Channels. J Phys Chem A. 2000;104: 1876–1889. doi:10.1021/jp993732q

14. Kovács M, Wang F, Hu A, Zhang Y, Sellers JR. Functional divergence of human cytoplasmic myosin II. Kinetic characterization of the non-muscle IIA isoform. J Biol Chem. 2003;278: 38132–38140. doi:10.1074/jbc.M305453200

15. Stam S, Alberts J, Gardel ML, Munro E. Isoforms confer characteristic force generation and mechanosensation by myosin II filaments. Biophys J. Biophysical Society; 2015;108: 1997–2006. doi:10.1016/j.bpj.2015.03.030

16. Hill A V. The possible effects of the aggregation of the molecule of hemoglobin on its dissociation curves. J Physiol. 1910;40: iv–vii. doi:10.1017/CBO9781107415324.004

17. Lin J. Divergence Measures Based on the Shannon Entropy. IEEE Trans Inf Theory. 1991;37: 145–151. doi:10.1109/18.61115
